# A multicomponent digital intervention to promote help-seeking for mental health problems and suicide in sexual and gender diverse young adults: A randomized controlled trial

**DOI:** 10.1371/journal.pmed.1004197

**Published:** 2023-03-06

**Authors:** Meng Han, Yinzhe Wang, Yanwen Zhang, Yuanyuan Wang, Jianjun Ou, Daixi Ren, Chengxi Cai, Kunxu Liu, Runan Li, Jin Han, Runsen Chen

**Affiliations:** 1 Vanke School of Public Health, Tsinghua University, Beijing, China; 2 Institute for Healthy China, Tsinghua University, Beijing, China; 3 University of Pennsylvania, Philadelphia, Pennsylvania, United States of America; 4 Key Laboratory of Brain, Cognition and Education Sciences, Ministry of Education, China; School of Psychology, Center for Studies of Psychological Application, and Guangdong Key Laboratory of Mental Health and Cognitive Science, South China Normal University, Guangzhou, China; 5 Department of Psychiatry, National Clinical Research Center for Mental Disorders, The Second Xiangya Hospital of Central South University, Changsha, China; 6 Black Dog Institute, University of New South Wales, Sydney, NSW, Australia; Addis Ababa University / King’s College London, ETHIOPIA

## Abstract

**Background:**

LGBTQ+ community’s higher susceptibility to worse mental health outcomes and more help-seeking barriers compared to the *cis*-heterosexual population. Despite the LGBTQ+ population facing higher mental health risks, there has been a dearth of research focusing on developing tailored interventions targeting them. This study aimed to assess the effectiveness of a digital multicomponent intervention in promoting help-seeking for mental health issues in LGBTQ+ young adults.

**Methods and findings:**

We recruited LGBTQ+ young adults aged between 18 and 29 who scored moderate or above on at least 1 dimension of the Depression Anxiety Stress Scale 21 and did not have help-seeking experiences in the past 12 months. Participants (*n* = 144) were stratified by gender assigned at birth (male/female) and randomly allocated (1:1 ratio) to the intervention or active control parallel condition by generating a random number table, so they were blinded to the intervention condition. All participants received online psychoeducational videos, online facilitator-led group discussions, and electronic brochures in December 2021 and January 2022, with the final follow-up in April 2022. The contents of the video, discussion, and brochure are help-seeking for the intervention group and general mental health knowledge for the control group. The primary outcomes were help-seeking intentions for emotional problems and suicidal ideation and attitudes toward seeking help from mental health professionals at the 1-month follow-up. The analysis was performed by including all participants based on their randomized group regardless of adherence to the protocol. A linear mixed model (LMM) was used for analysis. All models were adjusted for baseline scores. Chinese Clinical Trial Registry: ChiCTR2100053248.

A total of 137 (95.1%) participants completed a 3-month follow-up, and 4 participants from the intervention condition and 3 from the control condition did not complete the final survey. Compared with the control group (*n* = 72), a significant improvement was found in help-seeking intentions for suicidal ideation in the intervention group (*n* = 70) at post-discussion (mean difference = 0.22, 95% CI [0.09, 0.36], *p* = 0.005), 1-month (mean difference = 0.19, 95% CI [0.06, 0.33], *p* = 0.018), and 3-month follow-ups (mean difference = 0.25, 95% CI [0.11, 0.38], *p* = 0.001). There was also a significant improvement in the intervention condition on the help-seeking intention for emotional problems at 1-month (mean difference = 0.17, 95% CI [0.05, 0.28], *p* = 0.013) and 3-month follow-ups (mean difference = 0.16, 95% CI [0.04, 0.27], *p* = 0.022) compared with the control group. Participants’ depression and anxiety literacy and help-seeking encouragement related knowledge in intervention conditions showed significant improvements. There were no significant improvements in actual help-seeking behaviors, self-stigma toward seeking professional assistance, depression, and anxiety symptoms. No adverse events or side effects were observed. However, the follow-up time point was limited to 3 months which might not be long enough for drastic mindset and behavioral changes in help-seeking to occur.

**Conclusions:**

The current intervention was an effective approach in promoting help-seeking intentions, mental health literacy, and help-seeking encouragement-related knowledge. Its brief yet integrated intervention format could also be utilized in treating other imminent concerns confronted by LGBTQ+ young adults.

**Trial Registration:**

Chictr.org.cn, ChiCTR2100053248.

## Abstract 2

In a randomized controlled trial, Runsen Chen and colleagues investigate the impact of a multi-component digital intervention to promote help-seeking for mental health problems and suicide in sexual and gender diverse young adults.

## Author summary

**Why was this study done?:**

- Members of the LGBTQ+ community, compared to their *cis*-heterosexual counterparts, are less likely to seek help for mental problems due to minority stress, stigma, and difficulty in acquiring sexual- and gender-affirmative care.
- Findings from past studies suggest that psychoeducational videos, facilitator-led group discussions, and help-seeking brochures are all key influential factors for individuals’ help-seeking.
- Nevertheless, few studies have investigated the effectiveness of multicomponent approaches and digital online-based interventions targeted at LGBTQ+ young adults’ help-seeking behaviors.

**What did researchers do and find?:**

- **We conducted a single-blind parallel-group randomized control trial to evaluate the efficacy of a digital online-based multicomponent intervention in promoting help-seeking among LGBTQ+ young adults with mental health problems.**
- We included 144 participants recruited between December 3, 2021 and January 12, 2022 through online platforms who had no help-seeking experience within the past 12 months.
- We randomized 72 participants to the help-seeking promoting condition and compared them to 72 who received general health knowledge condition. The study was conducted over 5 months.
- Our findings indicated that an integrated intervention that combines psychoeducational videos, facilitator-led group discussions, and help-seeking brochures was effective in improving LGBTQ+ young adults’ general help-seeking intentions.

**What do these findings mean?:**

- Our study offers preliminary evidence that digital interventions can improve help-seeking for LGBTQ+ young adults with mental health difficulties.
- Integration of digital interventions by policymakers to care provided to LGBTQ+ young adults may help to improve help-seeking and mental health outcomes on a wider societal level.

## Introduction

The LGBTQ+ population is at greater risk than their *cis*-heterosexual counterparts for adverse mental health outcomes, including depression, anxiety disorders, and suicidality [1–5]. Such disparities are driven by stressors unique to the LGBTQ+ population [4,6,7]. For example, among various stressors, the family pressure faced by LGBTQ+ young people in China, exemplified in the parental generation’s implicit expectation of their LGBTQ+ offspring to conform to culturally ideal normative (e.g., heterosexual marriage and procreation), could be deemed as the most prevalent and pervasive, especially considering the key role of filial piety in traditional Chinese culture [8,9].

The minority stress model accentuates that the barriers resulting in the LGBTQ+ population’s reluctance to help-seeking can be categorized into distal and proximal stressors [6,10,11]. Findings from previous research indicate that distal stressors that heavily impact LGBTQ+ individuals’ externally based experiences contain the fear of being perceived negatively and the fear of being rejected by mental health professionals (MHPs) during treatments due to their implicit biases towards the LGBTQ+ community and their incompetency in offering LGBTQ+ affirmative care [6,10,12]; proximal stressors that profoundly influence LGBTQ+ individuals’ internally based experiences, in turn, are gradually shaped by abovementioned distal stressors and are constituted by over-reliance on self-coping, frightful perception of mental health conditions, and diffidence or insecurity when facing MHPs during treatments [6,10,12,13]. The combination of distal and proximal stressors contributes to the help-seeking stigma among LGBTQ+ individuals, manifested in their low help-seeking intentions and negative help-seeking attitudes (e.g., distrust in MHPs’ ethics and promise of confidentiality that is based on no solid evidence, one’s generalized prejudice, or hearsay) [14]. Besides the interpretation formulated based on the minority stress model, a number of studies also reiterated specific barriers that LGBTQ+ individuals face, such as the lack of financial resources for transgender individual’s gender transition or for LGB individuals’ mental health services, that could lower their help-seeking intentions, worsen their help-seeking attitudes, and reduce the likelihood of actual help-seeking behaviors [15,16].

To our best knowledge, despite much research on promoting help-seeking intentions, improving help-seeking attitudes, and encouraging actual help-seeking behaviors in the *cis*-heterosexual population, there is little research targeting the LGBTQ+ population that utilizes an integrated approach with the aim to promote all 3 outcomes, leaving a critical gap in the literature. Specifically, 1 randomized clinical trial that centered on promoting LGBTQ+ individuals’ help-seeking was conducted in a German setting, with its emphasis on difficulties related to “coming out” [17]. The study specifically used narratives from LGBTQ+ individuals who have been through the “coming-out” phase with the aim of alleviating their participants’ suicidal ideation, promoting help-seeking intention, reducing hopelessness, and promoting coping towards identity challenges. The study found that video narrative was effective in alleviating suicidal ideation and promoting help-seeking intentions (only with those who identify with the video), but the effects were short-lived. Another study that was centered on promoting help-seeking in the LGBTQ+ population was a randomized controlled trial that tested the effects of a game-based intervention, and the results showed, despite the intervention lowered the frequency of maladaptive behaviors (e.g., binge drinking and marijuana use), the study was not powered to find significant effects for help-seeking intentions, help-seeking self-efficacy, and help-seeking behaviors [18]. Furthermore, the target sample of the study included all LGBTQ+ individuals who have experienced bullying/cyberbullying victimization and was not specified on LGBTQ+ individuals who have mental health concerns, as it was not shown in its eligibility criteria [18].

It is an important objective to improve LGBTQ+ young adults help-seeking for mental health conditions. It is equally important to improve their attitude and intention toward help-seeking from both professionals and the general population [19]. Seeking help from professionals can effectively improve the mental health distress of the LGBTQ+ population and prevent suicide risk and self-injury. Nevertheless, considering (1) the LGBTQ+ population would often experience a strong stigma when seeking help for mental health issues; and (2) the lack of affirmative professionals specialized in LGBTQ+ care in China, it is difficult to seek professional help in many cases [20]. When professionals are not available, seeking help from friends, family members, and other parties in the general population can also help to lower suicide risks and mental health concerns [21]. Therefore, the present study shared a similar goal of nourishing LGBTQ+ young adults by improving the attitude or intention to seek help from both professionals and non-professional populations. Nevertheless, the target population and the intervention format between the previous and the present study were different. Compared to the previous study’s general LGBTQ+ population, the target population in the present study consisted of LGBTQ+ young adults with mental health conditions who had no help-seeking experience within the past 12 months, signaling increased clinical significance. Furthermore, regarding the intervention format, compared to the previous study’s sole concentration on utilizing self-disclosure videos, the present study focused on interaction and employed an integrated approach that was fully online and consisted of not only psychoeducational videos but also group discussions and help-seeking brochures.

The World Health Organization (WHO) postulates that mobile technology health interventions are featured by the effective utilization of mobile technologies to meet the unmet needs of traditional health service delivery [22]. As previously noted, past research suggests that the LGBTQ+ population is disadvantaged in treatments that they need, often being restrained by limited financial resources and reduced availability of both medical and mental health services [23–25]. Therefore, we utilized the digital online-based format (i.e., the entire intervention was conducted via a video conferencing platform that incurs no costs for participants) to conduct our intervention. Past studies have demonstrated the efficacy of utilizing remote online conferencing as an intervention format to increase adherence, promote engagement, and reduce dropout rates [26,27]. In addition, the online-based format has greater accessibility and extensive outreach compared to traditional face-to-face intervention delivery [26–28]. Previous research has shown that online help-seeking interventions effectively promoted help-seeking outcomes in the *cis*-heterosexual population, resulting in a gap in the literature that the current study aims to fill [29].

Given the absence of research in promoting the LGBTQ+ population’s help-seeking intentions and attitudes using a digital online-based multicomponent approach, the present study sought to test the potential efficacy of this intervention. We employed a control condition in which participants would undergo the same intervention process with the same online format and time length while no mental help-seeking contents (focus on sleep hygiene, healthy diet, and exercise) in the intervention condition were involved. This study examined the efficacy of the digital online-based multicomponent help-seeking intervention targeting the LGBTQ+ population by comparing results of primary outcomes (i.e., help-seeking intention and attitude) and secondary outcomes (i.e., actual help-seeking behaviors, depression, anxiety literacy, help-seeking stigma, and help-seeking encouragement-related knowledge) from participants in the intervention condition against the control condition.

## Methods

We conducted this single-blind parallel-group superiority randomized controlled trial to demonstrate the effectiveness of a brief digital online-based multicomponent intervention to promote help-seeking in LGBTQ+ young adults. The present study is reported in accordance with the Consolidated Standards of Reporting Trials (CONSORT) guideline (S1 CONSORT Checklist). This trial’s protocol was registered at the Chinese Clinical Trial Registry [30] (ChiCTR; identifier: ChiCTR2100053248) and has been published elsewhere [31].

### Study design and participants

Recruitment was online, with recruitment posters being released through multiple social media platforms such as WeChat, QQ, Dou ban, and Sina Weibo, which are popular in China. By scanning the quick response (QR) code on the advertisement poster, participants who were willing to join the study answered the screening questionnaire and provided their contact numbers. Participants aged between 18 and 29 years old who self-identified as a member of the LGBTQ+ community were eligible to participate if they were living in China, had stable internet connections, scored moderate or above on at least 1 dimension (cut-off values were 14 points for depression, 10 points for anxiety, and 19 points for stress) of the Depression Anxiety Stress Scale 21 [32,33], and did not have help-seeking experiences in the past 12 months (asked by a single question that whether they had sought for professional help in the past 12 months).

Participants were excluded if they met one or more of the following criteria: had a history of being diagnosed with psychotic disorder, such as schizophrenia; have had suicidal attempt(s) in the last 6 months; currently have severe suicide ideations; could not complete all assessments and intervention procedures in a quiet, undisturbed space; or had help-seeking experience(s) from MHPs in the past 12 months. Participants have a one-to-one telephone conversation with a researcher to confirm whether they meet the inclusion and exclusion criteria.

Participants who met the study criteria went through the one-to-one consent-informing phase with one of the researchers via phone again. The electronic consent was sent to participants, and pictures of the handwritten signed consent or e-signature consent were returned to the researchers. Participants were recruited in 2 time periods: 80 participants were enrolled in December 2021, and 64 participants were enrolled in January 2022. All enrolled participants provided informed consent in written format. Participants have the right to withdraw their informed consent without reason at any time during the intervention and follow-up period, and their data will not be used in the analysis.

### Randomization and masking

First, a random number table was generated by a researcher who was not involved in the intervention using IBM SPSS Statistics version 26.0. Each participant was assigned a unique number in the order of enrolling in the study. Then, the unique number of participants was separated from other information and sent to the researcher who generated the random number table, and this researcher, using the random number table, assigned participants to intervention or control condition. In this process, this researcher will not be exposed to other personal information of participants. Even though the recruitment of participants is divided into 2 periods, because the unified random number table generated in advance is used, the randomization process was guaranteed to be single-random.

A total of 144 participants who signed the informed consent form were stratified by gender assigned at birth (male/female) and then randomly allocated (1:1) to the experimental or control condition. Participants were masked to their group assignment, although they were informed of the existence of another group. The unified numbering procedure for all participants was used to minimize the possibility of participants knowing their groups according to the assigned number. Due to the nature of the present study, masking the study group facilitators was impossible. Nevertheless, we minimized the experimenter effect by having 3 group facilitators assigned to the experimental and control conditions, respectively.

### Procedures

Following randomization, participants were asked to complete the online baseline questionnaire and to choose their time slots to participate in the group discussion based on their schedules. Researchers then grouped participants according to their selected time slots. Each group, consisting of 4 to 6 participants, was assigned 1 researcher as the group facilitator to help participants complete all the intervention procedures. The entire intervention was conducted on an online video conferencing platform (Tencent Meeting, a conference software with functions very similar to ZOOM). Each participant received the encrypted online meeting link of one’s group and was notified to attend the meeting as scheduled. Due to the sudden changes in the schedules or dropouts, there were 2 groups that had only 3 participants.

This digital online-based intervention has incorporated 3 key therapeutic components, including (1) online animated psychoeducational videos (S1 Fig); (2) online facilitator-led group discussions (S2 Fig); and (3) electronic help-seeking brochures (S3 Fig/S4 Fig), with the efficacy of each proven by previous studies [34–38]. More specifically, animated psychoeducational videos have been shown to be more tone-neutral and acceptable than traditional psychoeducational videos [34,35,38]; according to the Interpersonal Psychotherapy (IPT) theory, facilitator-led group discussions have been recognized as an effective way to induce in-group empathy, promote attitude change, and advance positive thinking by facilitating group members’ self-disclosures [36]; the contents included in the help-seeking brochures were help-seeking encouragement, references to LGBTQ+ affirmative resources, and contact information of these resources, aligning with findings from previous research [37].

The current intervention was developed by the study team. Regarding the intervention videos, 5 modules in the intervention video were all presented from the perspective of LGBTQ+ populations, and the characters used were LGBTQ+ friendly. The intervention video also contains LGBTQ+-friendly doctors and practitioners in the psychology and psychiatry fields and 2 role models from the LGBTQ+ community (1 homosexual and 1 transgender). Regarding facilitator-led group discussions, structured interview guidelines were created by the supervisor of the project and were printed out during group discussions. During facilitator-led group discussions, 3 facilitators acquired participants’ verbal consent and introduced confidentiality to them. The help-seeking brochure was graphically designed by one of the research assistants. Its content included LGBTQ+-friendly knowledge and help-seeking resources. As mentioned in our study protocol [31], all materials prepared in the study were discussed and approved in the focus groups. The focus groups included 4 psychiatrists (average year working with LGBTQ+ was 3.6 years), 2 psychologists (average year working with LGBTQ+ was 7 years), 1 sociologist (average year working with LGBTQ+ was 3 years), and 5 LGBTQ+-friendly psychological consultants and psychotherapists (average year working with LGBTQ+ was 4 years). In addition, other researchers from UNSW Black Dog Institute were also involved in the development of the intervention. The development of the current intervention was based on previous research for the Chinese population [36,39]. For detailed descriptions of intervention contents for the experimental and the control conditions, please refer to our published study protocol [31].

Regarding the intervention process, group facilitators delivered this brief digital online-based multicomponent intervention by leading participants in completing the following 3 essential steps: (1) watching the entire 15-min intervention video (i.e., videos in both conditions were of the same length) with other group members in the online conference room; (2) actively participating in the structured discussions regarding intervention video and electronic psychoeducational brochure with other participants in the online conference room; and (3) freely accessing and reviewing the intervention video and electronic brochure post-discussion via web link given.

All 3 facilitators of the current study had a master’s degree in psychology and received 7 times internal training (2 to 3 h for each training) from 2 senior researchers (a doctoral degree in psychology and a doctoral degree in psychiatry research) before the start of the study (see investigators training section in appendix). The intervention process was recorded and checked to ensure intervention fidelity. Two senior researchers listened to the recordings of the intervention procedure independently and evaluated the fidelity of facilitators. The fidelity evaluation using a list developed by the research team, which included 6 items assessing video fluency, facilitators’ involvement, facilitators’ friendliness, participants’ activity, participants’ emotional involvement, and the overall evaluation of the process. Each item scored 0 to 5, using the average score of 6 items to evaluate the completion of a group discussion. Two senior researchers each listened to 6 recordings, including 3 intervention group recordings and 3 control group recordings from 3 facilitators, respectively. The average fidelity scores given by the 2 senior researchers were from 4.58 to 4.67 (full score of 5 points).

Intervention videos for both conditions were safely stored in a password-protected online server at Wenjuanxing, a Chinese online survey platform (i.e., similar to Qualtrics.com but can store videos and contents for guest viewing and downloading through the online address and password sent out by the researchers), during the course of the study. The electronic psychoeducational brochures for both conditions were sent out by researchers to participants directly for them to save and view at any moment during their free time. After group discussion and a brief introduction of the brochure, participants in the experimental and the control conditions were asked to complete an online post-discussion questionnaire. Participants were also asked to complete online questionnaires at 1-month and 3-month follow-ups. If participants did not reply within 5 days of receiving the online questionnaire link, they would receive a reminder text asking them to complete the questionnaire as soon as possible. If participants still did not reply within 2 days after the reminder, they would be marked as lost to follow up at this time point. Participants in the control condition also received the intervention video and psychoeducational brochure same as the intervention condition after the 3-month follow-up.

To ensure the safety of participants, we have developed a directory consisting of a list of psychiatric hospitals and their appointment registration methods in many cities in China, online and offline psychological intervention resources, and a list of the main Chinese crisis helplines. For participants who were excluded due to psychotic disorder or high suicide risk in the recruitment stage, we provided them with the directory and advised them to seek professional help. For participants involved in this study, whether in the intervention condition or control condition, the end of the brochures they received was also attached with the same directory mentioned above so they can get professional help at any time through this information if they need it. Ethical approval was granted by the Institution Review Board of Tsinghua University (Approval No. 20210146).

### Outcomes

The primary outcomes were participants’ changes in general help-seeking intentions for emotional problems and suicidal ideation and attitudes toward seeking help from MHPs between baseline and 1-month follow-up. The general help-seeking intentions were measured by the General Help-Seeking Questionnaire (GHSQ), the validity of which in Chinese participants had been validated by previous research [39]. GHSQ consisted of 22 items divided into 2 subscales (11 items for emotional problems and 11 items for suicidal ideation) and was scored on a 4-point Likert scale, with a higher score indicating a stronger intention to seek help. Participants’ attitudes toward seeking help from MHPs were measured by the Attitudes Toward Seeking Professional Psychological Help Scale Short Form (ATSPPH-SF), the validity of which had also been validated in Chinese participants by previous research [40]. The validated Chinese version of the ATSPPH-SF consisted of 10 items on a 4-point Likert scale, with a higher score indicating a more positive attitude toward seeking professional help [40]. Since GHSQ and ATSPPH-SF measured different aspects of help-seeking for mental health issues, and both are the focus of intervention in the current study, we defined that they are parallel primary outcomes.

Participants’ changes in general help-seeking intentions for emotional problems and suicidal ideation and attitudes toward seeking help from MHPs between baseline and post-discussion and 3-month post-intervention were defined as the secondary outcomes. Other secondary outcomes included participants’ actual help-seeking behaviors measured by the Chinese validated version of the Actual Help-Seeking Questionnaire (AHSQ) [41], participants’ depression and anxiety literacy measured by the Depression and Anxiety Literacy Questionnaire (D-A-Lit) [42,43], participants’ self-stigma toward seeking professional assistance measured by the Chinese validated version of Self-Stigma of Seeking Help (SSOSH) Scale [44], and participants’ help-seeking encouragement-related knowledge (i.e., knowledge in encouraging others for help-seeking) measured by the Chinese validated version of the Help-Seeking Encouragement-Related Knowledge Scale [39].

Since the GHSQ and AHSQ have the “not applicable” option, these 2 variables used the average value of valid items for analysis, while other variables used the total score. According to previous research, we did the additional analysis of the GHSQ by dichotomizing responses into “0” for “Highly unlikely” and “Unlikely,” and “1” for “Highly likely” and “Likely” [36].

### Statistical analysis

Based on previous research, moderate effect sizes of help-seeking intentions (Cohen’s d = 0.53) and help-seeking attitudes (Cohen’s d = 0.58) were expected for the present study’s primary outcomes [45]. The sample size was conservatively estimated (*n* = 144), assuming 20% attrition and a 1:1 allocation ratio during randomization [45], which would provide 80% power to detect an effect size of 0.53 at the α level of 0.05 for each primary outcomes.

Analyses for both primary and secondary outcomes were done from the following time points, respectively: (1) post-discussion; (2) 1-month follow-up; and (3) 3-month follow-up, with the exception of actual help-seeking behaviors of participants, which did not include the post-discussion measurement time point. The analysis was performed by including all participants based on their randomized group regardless of adherence to the protocol. The missing data was not processed since it is less than 5%. We used linear mixed model (LMM) to analyze the difference between the intervention effect on the primary outcomes and secondary outcomes, with the baseline score included as a covariate. The fixed effects included intervention, time of assessment, and interactions. The random intercepts of participants were considered in the random effects of the models, and all models were estimated using maximum-likelihood estimation methods.

For the additional analysis of AHSQ and GHSQ, each item was analyzed independently by using the generalized linear mixed model (GLMM), and the results were presented in the Appendix (S1 Table). In addition, we used the independent sample *t* test as a measure to investigate whether there were differences in participants’ evaluations of intervention quality between the intervention and control conditions. All statistical analyses were performed with IBM SPSS Statistics version 26.0 and R software version 4.2. The significance level of each analysis will be set to 0.05 while simultaneously reporting a 95% confidence interval. Considering the accuracy and robustness of the results, we have modified the analytical procedures mentioned in our protocol. Firstly, considering the multiple primary outcomes leading to a higher type I error rate, we used the Bonferroni correction method to adjust the *P* value of the 3 primary outcomes. Furthermore, we added a sensitivity analysis for all outcomes in the Appendix (S2 Table), which presented results using a mixed linear model that conducted a post hoc analysis calculating the difference from baseline. This trial was registered at www.chictr.org.cn, with the registration number ChiCTR2100053248.

## Results

**Fig 1:**
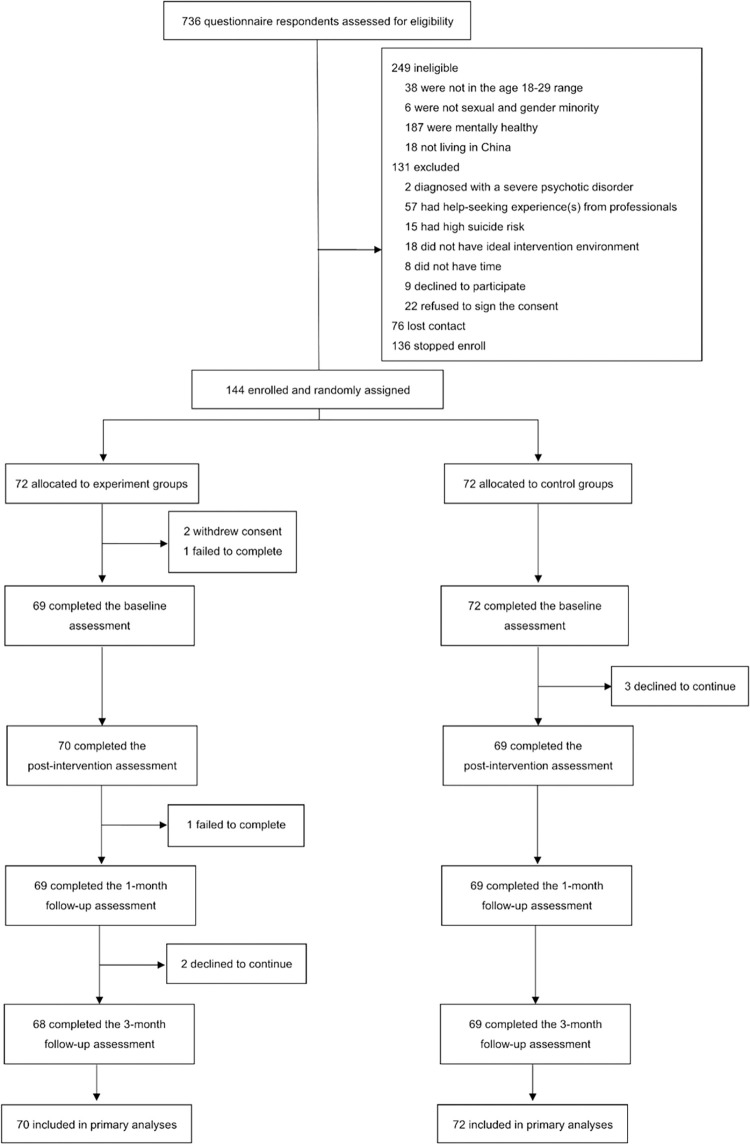
Flow diagram detailing recruitment and randomization of trial participants.

Participants were recruited between December 3, 2021 and January 12, 2022. A total of 736 questionnaire respondents were assessed for eligibility, among which 592 respondents were either ineligible, met the exclusion criteria, lost contact, or stopped enrolling (Fig 1). After signing the consent, participants were notified that they were officially enrolled in the study and assigned a unique number. When the present study reached its planned number of participants (*n* = 144), recruitment was discontinued. During the post-discussion phase, 2 participants in the intervention condition withdrew their written consent and dropped out of the study, leaving 142 participants for primary analyses, and 139 out of 142 participants completed the intervention procedure, with 3 participants in the control condition dropping out before the start of the intervention. One participant in the intervention condition did not successfully submit baseline data, and 138 (95.8%) of 144 participants had successfully completed the 1-month follow-up questionnaire, and 137 (95.1%) of 144 participants had completed the 3-month follow-up questionnaire (Fig 1). The mean length of follow-up of all participants was 85.64 days (SD = 2.19), ranging from 83 to 93 days. The mean age of the participants was 21.84 (SD = 2.79) for the intervention condition and 22.49 (SD = 2.78) for the control. The gender identity, educational level, and other demographic characteristics were similar between conditions which were shown in Table 1.

**Table 1:** Participants’ demographics and mental health characteristics at the screening by group.

	Total sample (*n* = 142)	Intervention (*n* = 70)	Control (*n* = 72)
Age (years, 18–29)	22.17 (2.80)	21.84 (2.79)	22.49 (2.78)
Gender identity			
Cisgender male	33 (23.2)	17 (24.3)	16 (22.2)
Cisgender female	39 (27.5)	18 (25.7)	21 (29.2)
Transgender man	17 (12.0)	7 (10.0)	10 (13.9)
Transgender woman	18 (12.7)	6 (8.6)	12 (16.7)
Nonbinary or genderqueer	29 (20.4)	17 (24.3)	12 (16.7)
Others	6 (4.2)	5 (7.1)	1 (1.4)
Sexual orientation			
Bisexual	26 (18.3)	12 (17.1)	14 (19.4)
Gay/lesbian	51 (35.9)	24 (34.3)	27 (37.5)
Heterosexual or straight	16 (11.3)	4 (5.7)	12 (16.7)
Pansexual	37 (26.1)	20 (28.6)	17 (23.6)
Others (include asexual)	12 (8.5)	10 (14.3)	2 (2.8)
Educational*			
Middle school/high school	8 (5.6)	4 (5.7)	4 (5.6)
Bachelor/junior college	104 (73.2)	50 (71.4)	54 (75.0)
Master or higher	29 (20.4)	15 (21.4)	14 (19.4)
Ethnicity*, Han ethnic group	131 (92.3)	64 (91.4)	67 (93.1)
Religious belief*, no	132 (93.0)	65 (92.9)	67 (93.1)
Type of family grow up in*			
Nuclear	100 (70.4)	52 (74.3)	48 (66.7)
Extended	21 (14.8)	8 (11.4)	13 (18.1)
Single-parent	12 (8.5)	6 (8.6)	6 (8.3)
Others	8 (5.6)	3 (4.3)	5 (6.9)
Only-child*, yes	90 (63.4)	41 (58.6)	49 (68.1)
Annual family income, ¥*			
Less than 6,000	10 (7.1)	5 (7.1)	5 (6.9)
6,000–14000	26 (18.3)	10 (14.3)	16 (22.2)
14,000–23,000	24 (16.9)	10 (14.3)	14 (19.4)
23,000–36,000	28 (19.7)	12 (17.1)	16 (22.2)
36,000–70,000	28 (19.7)	18 (25.7)	10 (13.9)
70,000 or more	25 (17.6)	14 (20.0)	11 (15.3)
Depression			
Moderate	54 (38.0)	29 (41.4)	25 (34.7)
Severe	29 (20.4)	14 (20.0)	15 (20.8)
Extremely severe	18 (12.7)	9 (12.9)	9 (12.5)
Anxiety			
Moderate	54 (38.0)	25 (35.7)	29 (40.3)
Severe	39 (27.5)	21 (30.0)	18 (25.0)
Extremely severe	30 (21.1)	17 (24.3)	13 (18.1)
Stress			
Moderate	36 (25.4)	23 (32.9)	13 (18.1)
Severe	26 (18.3)	10 (14.3)	16 (22.2)
Extremely severe	6 (4.2)	4 (5.7)	2 (2.8)

Data are mean (SD) or n (%).

*One participant in the experimental condition had missing data for these variables.

**Fig 2:**
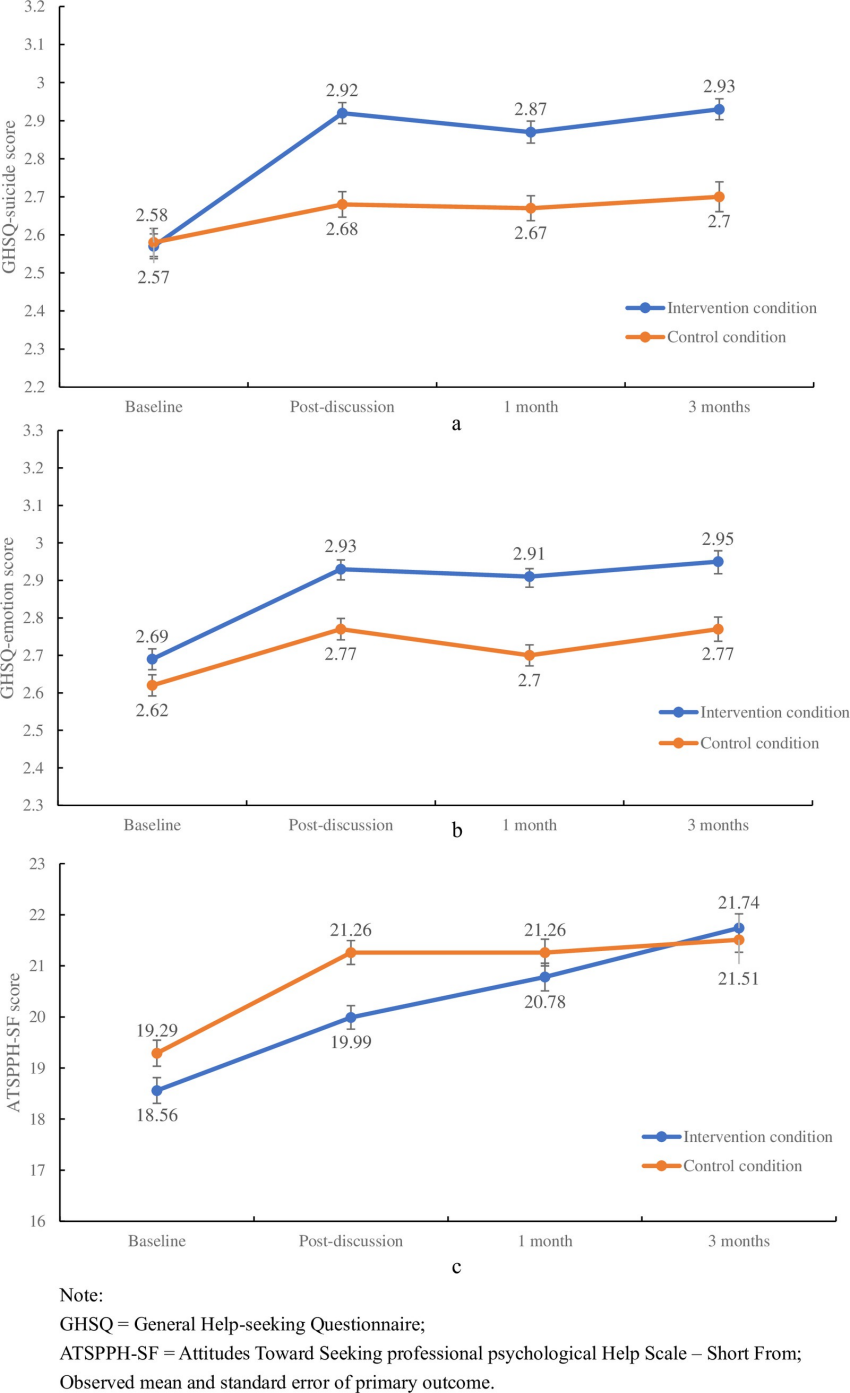
Line charts of primary outcomes. (**a**) shows the results of help-seeking intention of suicidal ideation. (**b**) shows the results of help-seeking intention of emotional issues. (**c**) shows the results of attitudes toward seeking help from MHPs.

For primary outcomes (Table 2), results from the LMM indicated that, compared with the control condition, participants in the intervention condition showed a significant improvement in the help-seeking intention of suicidal ideation at 1-month follow-up (mean difference = 0.19, 95% CI [0.06, 0.33], *p* = 0.018) (Fig 2a). For the help-seeking intention of emotional issues, the intervention effect showed a significant improvement at 1-month follow-ups (mean difference = 0.17, 95% CI [0.05, 0.28], *p* = 0.013) (Fig 2b). However, results showed that there was no significant improvement in attitudes toward seeking help from MHPs (Fig 2c).

**Table 2:** Results from the linear mixed model.

	Observed estimates				Model-based estimates			
	Intervention condition		Control condition		Intervention condition	Control condition	Mean difference ^a^ (95% CI)	*P* value
	N	Mean (SD)	N	Mean (SD)	Mean (95 CI%)	Mean (95 CI%)		
**Primary outcomes**								
**GHSQ**								
GHSQ-emotion score ^b^^,^ ^c^								
Baseline	68	2.69 (0.48)	72	2.62 (0.50)	-	-		
Post-discussion	70	2.93 (0.43)	69	2.77 (0.50)	2.91 (2.82, 2.99)	2.79 (2.71, 2.86)	0.12 (0.01, 0.23)	0.104 ^f^
1 month ^e^	69	2.91 (0.37)	69	2.70 (0.49)	2.89 (2.81, 2.97)	2.72 (2.64, 2.80)	0.17 (0.05, 0.28)	0.013 ^f^
3 months	68	2.95 (0.50)	69	2.77 (0.56)	2.94 (2.86, 3.02)	2.79 (2.71, 2.87)	0.16 (0.04, 0.27)	0.022 ^f^
GHSQ-suicide score ^b^^,^ ^c^								
Baseline	68	2.57 (0.57)	70	2.58 (0.65)	-	-		
Post-discussion	69	2.92 (0.48)	66	2.68 (0.57)	2.90 (2.81, 3.00)	2.68 (2.59, 2.78)	0.22 (0.09, 0.36)	0.005 ^f^
1 month ^e^	67	2.87 (0.50)	66	2.67 (0.56)	2.87 (2.77, 2.97)	2.68 (2.58, 2.77)	0.19 (0.06, 0.33)	0.018 ^f^
3 months	68	2.93 (0.48)	67	2.70 (0.67)	2.94 (2.84, 3.04)	2.69 (2.60, 2.79)	0.25 (0.11, 0.38)	0.001 ^f^
**ATSPPH-SF score** ^c^								
Baseline	69	18.56 (4.40)	72	19.29 (4.56)	-	-		
Post-discussion	70	19.99 (4.05)	69	21.26 (4.09)	20.18 (19.28, 21.07)	21.04 (20.14, 21.93)	−0.86 (−2.14, 0.42)	0.563 ^f^
1 month ^e^	69	20.78 (4.74)	69	21.26 (4.58)	20.91 (20.01, 21.93)	21.04 (20.14, 21.93)	−0.13 (−1.42, 1.15)	1.000 ^f^
3 months	68	21.74 (4.80)	69	21.51 (4.29)	21.78 (20.88, 22.69)	21.29 (20.39, 22.18)	0.50 (−0.79, 1.79)	1.000 ^f^
**Secondary outcome**								
**AHSQ score** ^c^								
Baseline	69	0.46 (0.21)	72	0.41 (0.18)	-	-		
1 month ^e^	69	0.48 (0.22)	69	0.44 (0.22)	0.46 (0.42, 0.49)	0.46 (0.42, 0.50)	−0.003 (−0.06, 0.05)	0.913
3 months	68	0.49 (0.21)	69	0.44 (0.21)	0.47 (0.43, 0.51)	0.46 (0.42, 0.50)	0.01 (−0.05, 0.06)	0.748
**Depression-Anxiety-Lit**								
Depression-Lit score ^c^								
Baseline	69	13.38 (3.37)	72	12.90 (3.17)	-	-		
Post-discussion	70	13.76 (3.46)	69	12.12 (3.59)	13.63 (12.97, 14.30)	12.38 (11.72, 13.04)	1.25 (0.31, 2.20)	0.010
1 month ^e^	69	14.38 (3.45)	69	11.96 (4.11)	14.11 (13.44, 14.77)	12.22 (11.56, 12.89)	1.88 (0.93, 2.83)	<0.001
3 months	68	14.01 (3.62)	69	11.93 (4.35)	13.74 (13.07, 14.41)	12.19 (11.53, 12.86)	1.55 (0.60, 2.50)	0.002
Anxiety-Lit score ^c^								
Baseline	69	12.06 (2.82)	72	12.04 (2.94)	-	-		
Post-discussion	70	13.37 (3.25)	69	11.86 (3.28)	13.38 (12.72, 14.04)	11.88 (11.22, 12.54)	1.50 (0.56, 2.44)	0.002
1 month ^e^	69	14.01 (3.29)	69	11.94 (3.56)	13.92 (13.26, 14.59)	11.97 (11.31, 12.63)	1.96 (1.01, 2.90)	<0.001
3 months	68	13.91 (3.45)	69	11.91 (3.94)	13.90 (13.23, 14.56)	11.94 (11.28, 12.60)	1.96 (1.01, 2.90)	<0.001
**SSOSH score** ^d^								
Baseline	69	24.08 (6.35)	72	23.17 (5.28)	-	-		
Post-discussion	70	23.19 (5.27)	69	21.83 (5.67)	22.81 (21.77, 23.86)	22.15 (21.11, 23.20)	0.66 (−0.84, 2.16)	0.386
1 month ^e^	69	23.22 (6.10)	69	22.96 (5.80)	22.87 (21.82, 23.92)	23.28 (22.24, 24.33)	−0.42 (−1.92, 1.08)	0.585
3 months	68	22.75 (6.20)	69	22.14 (5.38)	22.53 (21.48, 23.59)	22.47 (21.42, 23.52)	0.06 (−1.44, 1.57)	0.934
**HSERK score** ^c^								
Baseline	69	32.94 (4.68)	72	33.19 (3.92)	-	-		
Post-discussion	70	34.26 (4.08)	69	33.32 (4.17)	34.50 (33.64, 35.35)	33.20 (32.34, 34.05)	1.30 (0.08, 2.52)	0.037
1 month ^e^	69	34.32 (4.44)	69	33.14 (4.55)	34.46 (33.61, 35.33)	33.02 (32.17, 33.88)	1.44 (0.22, 2.67)	0.021
3 months	68	35.04 (4.28)	69	33.59 (3.95)	35.09 (34.23, 35.96)	33.47 (32.62, 34.33)	1.62 (0.39, 2.85)	0.010
**DASS21**								
DASS21-depreesion score ^d^								
Baseline	69	19.22 (10.15)	72	17.06 (10.19)	-	-		-
Post-discussion	70	18.00 (10.45)	69	16.12 (10.28)	17.24 (15.46, 19.02)	16.79 (15.01, 18.57)	0.45 (−2.09, 2.99)	0.728
1 month ^e^	69	16.29 (9.73)	69	14.46 (10.94)	15.59 (13.80, 17.38)	15.14 (13.36, 16.92)	0.45 (−2.10, 3.00)	0.730
3 months	68	17.76 (10.72)	69	17.25 (9.54)	17.05 (15.25, 18.85)	17.92(16.14,19.70)	−0.87 (−3.43, 1.69)	0.504
DASS21-anxiety score ^d^								
Baseline	69	16.12 (9.11)	72	13.94 (8.44)	-	-		-
Post-discussion	70	16.29 (9.98)	69	14.49 (8.11)	15.30 (13.70, 16.91)	15.14 (13.54, 16.75)	0.16 (−2.14, 2.46)	0.889
1 month ^e^	69	14.03 (8.97)	69	12.72 (8.08)	13.17 (11.55, 14.79)	13.37 (11.77, 14.98)	−0.20 (−2.51, 2.10)	0.863
3 months	68	14.94 (9.21)	69	14.49 (8.71)	14.35 (12.73, 15.98)	15.14 (13.54, 16.75)	−0.79 (−3.10, 1.52)	0.503
DASS21-stress score ^d^								
Baseline	69	22.58 (9.24)	72	20.64 (9.75)	-	-		-
Post-discussion	70	21.60 (9.19)	69	20.64 (10.16)	20.86 (18.99, 22.73)	21.17 (19.31, 23.04)	−0.32 (−2.98, 2.35)	0.816
1 month ^e^	69	20.43 (9.33)	69	17.54 (10.57)	19.73 (17.85, 21.61)	18.07 (16.21, 19.94)	1.66 (−1.02, 4.34)	0.223
3 months	68	20.79 (8.69)	69	20.32 (10.03)	20.23 (18.34, 22.12)	20.86 (18.99, 22.72)	−0.62 (−3.31, 2.06)	0.648

^a^ All models adjusting baseline scores.

^b^ Some participants chose “not applicable” in the whole questionnaire.

^c^ Higher values correspond to better outcomes.

^d^ Higher values correspond to worse outcomes.

^e^ Primary time point.

^f^ *P* value was adjusted by the Bonferroni-corrected method (3 × *P* value), level of significance still was *P* < 0.05.

GHSQ, General Help-Seeking Questionnaire; ATSPPH-SF, Attitudes Toward Seeking Professional Psychological Help Scale-Short Form; AHSQ, Actual Help-Seeking Questionnaire; Depression-Lit, Depression Literacy Questionnaire; Anxiety-Lit, Anxiety Literacy Questionnaire; SSOSH, Self-Stigma of Seeking Help Scale; HSERK, Help-Seeking Encouragement Related Knowledge Scale; DASS21, Depression Anxiety and Stress Scale 21.

For secondary outcomes, results showed that compared with the control condition, participants in the intervention condition had a significant improvement in the help-seeking intention of suicidal ideation at post-discussion (mean difference = 0.22, 95% CI [0.09, 0.36], *p* = 0.005) and 3-month follow-up (mean difference = 0.25, 95% CI [0.11, 0.38], *p* = 0.001). For the help-seeking intention of emotional issues, the intervention effect showed a significant improvement at 3-month follow-ups (mean difference = 0.16, 95% CI [0.04, 0.27], *p* = 0.022) and no significant improvement at post-discussion (mean difference = 0.12, 95% CI [0.01, 0.23], *p* = 0.104).

For other secondary outcomes, there was no significant improvement in help-seeking behaviors and professional help-seeking stigma. Regarding the results of depression and anxiety literacy, participants in the intervention condition showed significantly improved depression and anxiety literacy at post-discussion (mean difference = 1.25, 95% CI [0.31, 2.20], *p* = 0.010; mean difference = 1.50, 95% CI [0.56, 2.44], *p* = 0.002) and 1-month follow-up (mean difference = 1.88, 95% CI [0.93, 2.83], *p* < 0.001; mean difference = 1.96, 95% CI [1.01, 2.90], *p* < 0.001). It was also worth noting that such intervention effect remained strongly significant at the 3-month follow-up (mean difference = 1.55, 95% CI [0.60, 2.50], *p* = 0.002; mean difference = 1.96, 95% CI [1.01, 2.90], *p* < 0.001). Furthermore, participants’ help-seeking encouragement-related knowledge had significant improvement at post-discussion, 1-month follow-up, and 3-month follow-up (mean difference = 1.30, 95% CI [0.08, 2.52], *p* = 0.037; mean difference = 1.44, 95% CI [0.22, 2.67], *p* = 0.021; mean difference = 1.62, 95% CI [0.39, 2.85], *p* = 0.010).

Besides, no differences in mental health (depression, anxiety, and stress) were observed between conditions in this study (Table 2). The results of intervention quality evaluated by participants showed that there were no differences between the intervention and control conditions (S3 Table). Results from follow-up evaluations for study participants also suggested high satisfaction rates for the study as a whole.

## Discussion

This randomized controlled trial aimed to examine the efficacy of a digital online-based multicomponent intervention promoting help-seeking in LGBTQ+ young adults with mental health problems. Compared to the control condition, participants who received the intervention showed improvements in general help-seeking intentions for both emotional problems and suicidal ideations. The significant differences at the primary time points (1-month follow-up) prove that the intervention program of the current study is effective. The mean differences between groups of help-seeking intention between baseline and 1-month follow-up were 0.17 for emotional problems and 0.19 for suicidal ideation. Compared with other similar studies [46,47], the mean differences of the current study at 1-month are significant and relatively higher, which indicates that the intervention program in this study can effectively improve the participants’ help-seeking intention at 1-month follow-up. The improvement in the help-seeking intention persisted during the 3-month follow-up, indicating that the effectiveness of the intervention can be better maintained. Additionally, participants who received the intervention showed significant improvements in depression and anxiety literacy at post-discussion, 1-month follow-up, and 3-month follow-up. Furthermore, participation in this intervention also significantly promoted participants’ help-seeking encouragement-related knowledge at post-discussion, 1-month follow-up, and 3-month follow-up. These findings underscore the promising potential of a brief, digital, online-based, yet multicomponent intervention as a practical option for ameliorating general help-seeking intentions and advancing depression and anxiety literacy in the LGBTQ+ population.

There is no cut-off value of the current scales used to assess the intentions of help-seeking [39,47]. We believe that future research should develop a validated scale, scores of which could reflect the impact of different levels of help-seeking intention on help-seeking behavior, to explore the correlation between the help-seeking intention and the actual help-seeking behavior. Nevertheless, these observed effects in the current study also showed that a lightweight psychological intervention could already play a role in improving the help-seeking intention in the LGBTQ+ population, underscoring the fact that such a highly cost-effective intervention format should be applied more extensively in LGBTQ+ health promotion campaigns. Realizing that one has mental health problems and need help is the first step to get effective mental health improvement. The results in our study showed that the intervention not only increased the participants’ mental health literacy, but also improved their intention to seek help, which means that the intervention program effectively help them take the first step to improve their mental health.

Aside from the improvements mentioned above, the intervention did not significantly promote participants’ professional help-seeking attitude and reduce participants’ help-seeking stigma. One potential explanation of these findings could be that the intricate connection between professional help-seeking attitude and help-seeking stigma has formulated a vicious cycle, blocking the intervention effect. Previous research indicated that help-seeking from LGBTQ+ individuals to professionals was deemed undesirable by the LGBTQ+ community in China due to the common idea embedded in the community culture that “help from each other (peer support)” could be more helpful compared to taking the risk of facing invalidation, discrimination, and prejudice to meet non-affirmative professionals, contributing to negative professional help-seeking attitudes and less motivation to seek help when in need [25,37,48–51]. Such a norm of seeking help from each other instead of professionals within the community could be the result of MHPs’ lack of sufficient training on LGBTQ+-affirmative care in China [52].

We also found the scores of primary outcomes of participants in the control condition increased over time. One possible explanation is that participants in the control condition have the placebo effect. That is, participants think they have accepted effective intervention, so they gave an increased score, which was also the effect of single-blinding. In addition, for the protection of participants at risk of suicide, we added a directory including 3 kinds of safety resources at the end of both brochures received by participants in the control condition and intervention condition. The increase in the help-seeking score of participants in the control condition may be due to this directory. With the purpose of avoiding the interference of these confounding factors, the current study was designed as a randomized controlled trial to verify the effectiveness of the intervention contents. Therefore, the impact of the directory on help-seeking was balanced between participants in 2 conditions.

Based on the structured discussion outline of the intervention condition (S2 Fig), participants in the intervention condition shared their feelings, new understanding of their emotional issues, and barriers to help-seeking after watching the intervention video. The sharing of experiences and feelings may lead other participants with similar experiences or feelings to feel that they are not alone and that their experiences are not unique [53]. This may reduce the stigma of participants. Then, participants shared ideas on how to overcome the barriers to help-seeking and how to make good use of the resources mentioned in the video and brochure. These discussions can help them better understand the content of the video and brochure and use the resources in them to seek help independently after discussion. Although the facilitators turned on audio and video during the discussion, most participants merely used audio during the discussion. Without seeing each other, participants are limited to focusing on the audio discussion and understanding the contents of the intervention video and brochure rather than interacting with each other. In addition, the group discussion took place in an online conferencing group only once, and the duration of the group discussion was only about 30 min when excluding the duration of watching videos and answering questionnaires. Based on these, we believe that the group has not yet formed a stable group, and the interaction between participants was limited.

One of the strengths of the current study was the willingness of participants to trust the researchers and disclose personal information such as educational background and family income. The potential reason for this is that, at the informed consent stage before the research, the researchers introduced the confidential content in the informed consent to the participants in detail, which also promoted the participants’ trust in the researcher so that they were willing to authentically answer their personal information. Considering the effectiveness of the primary outcomes in the current study and the high completion rate of participants, we believe that the digital online-based multicomponent intervention (including psychoeducational videos, online facilitator-led group discussions, and informative electronic help-seeking brochures) could also be applied to other general populations, so as to effectively promote help-seeking for mental health.

There were several other strengths from an implementation standpoint that needed to be addressed. First, the present intervention was conducted with high fidelity and completed with low dropout rates. Three group facilitators have received fidelity training on delivering this evidence-based intervention pre-intervention and passed the fidelity assessment examining adherence to study protocol, quality of delivery, and participant responsiveness with high fidelity ratings. Second, under the benefits of high fidelity, the completion rate was extremely high (95%), which was much higher compared to the average completion rate (80%) for most randomized controlled trials, suggesting that not only did we precisely and accurately implement intervention procedures as planned but also the high level of urgency of such interventions for the LGBTQ+ population [54]. The reasons for the high completion rate may include that: (1) all participants were contacted by a researcher in the way of one-to-one message, so when the participants failed to answer the questionnaires, the researcher would remind them one more time. (2) The participants’ educational level is relatively high, which also makes it easier for them to comply with the commitment to complete the research. Third, our intervention did not necessarily require the participation of MHPs. Once our intervention implementation process has been standardized and manualized in the near future, trainees who have undergone standard training based on our protocol could be expected to be able to carry out similar results. This will promote more LGBTQ+ young adults’ help-seeking intentions and mental health literacy, especially those who live in areas lacking LGBTQ+ mental health resources.

The primary limitation is that this study did not effectively promote participants’ actual help-seeking behaviors. The potential explanation could be the inference of the particular time period chosen to conduct both the present study and its post-intervention follow-ups. Chinese New Year (i.e., the Chinese Spring Festival) has always been the most celebrated national holiday in China, during which families, friends, and relatives reunite with each other. However, due to lower levels of both personal privacy and security arising from large family gatherings, it could also be the least possible occasion in which LGBTQ+ young adults would risk revealing their identities for help-seeking, subsequently blocking their actual help-seeking behaviors. Together with the impact of the pandemic (i.e., Coronavirus Disease 2019), even if they intended to seek help, significantly reduced availability and accessibility of resources was a difficulty they must overcome. The second limitation of this study was that the present study was limited to 3-month follow-ups, which might not be long enough for drastic mindset and behavioral changes in help-seeking to occur. Future studies should extend the follow-up time. The third limitation was that half of the participants of the current sample were transgender, nonbinary, genderqueer, or questioning population. This is because the promotion of online recruitment advertising of this study has been promoted by many LGBTQ+ community organizers. Online posters have been released through social media platforms, such as WeChat or QQ groups, which are popular in the Chinese LGBTQ+ community. Since our study received recognition from community organizers in the LGBTQ+ community, especially organizers from the transgender community, transgender young adults who usually could not be reached via posters and online research participation campaigns expressed their interest in participating in our study, explaining why there were more transgender participants in our study.

The present study strives to examine the efficacy of the digital online-based multicomponent intervention aiming to promote help-seeking in LGBTQ+ young adults. The intervention was found to be an effective integrated model for enhancing LGBTQ+ young adults’ general help-seeking intentions and promoting their depression and anxiety literacy. Although the clinical significance of the present study’s findings warrants future research, the results of the present study add to an emerging breadth of evidence indicating that a brief, digital, online-based, and multicomponent intervention holds great potential in downscaling currently existing mental health barriers faced by the LGBTQ+ population.

## Supporting information

S1 CONSORT Checklist CONSORT 2010 checklist of information to include when reporting a randomized trial. (DOCX)

S1 Fig Samples of the intervention video. (TIF)

S2 Fig The structured discussion outline of the intervention condition. (TIF)

S3 Fig Samples of the help-seeking brochure. (TIF)

S4 Fig Samples of the help-seeking brochure (English-translation). (TIF)

S1 Table Results from the generalized mixed linear model. (DOCX)

S2 Table Sensitivity Analyses of the linear mixed model. (DOCX)

S3 Table Results of the intervention evaluation. (DOCX)

S1 Text Items to include when reporting a randomized trial in a journal or conference abstract. (DOCX)

S1 Protocol Protocol. (DOCX)

S1 Data Outcome data in the main article. (CSV)

S2 Data Outcome data in supporting information. (CSV)

## Abbreviations

AHSQ: Actual Help-Seeking Questionnaire
ATSPPH-SF: Attitudes Toward Seeking Professional Psychological Help Scale Short Form
DASS: Depression Anxiety and Stress Scale
GHSQ: General Help-Seeking Questionnaire
GLMM: generalized linear mixed model
HSERK: Help-Seeking Encouragement Related Knowledge Scale
IPT: Interpersonal Psychotherapy
LMM: linear mixed model
MHP: mental health professional
QR: quick response
SSOSH: Self-Stigma of Seeking Help
WHO: World Health Organization
