# Supplementary figures and images for: A multicomponent digital intervention to promote help-seeking for mental health problems and suicide in sexual and gender diverse young adults: A randomized controlled trial
Source: PLoS Med. 2023 Mar 6;20(3):e1004197. doi: 10.1371/journal.pmed.1004197 (PMC10027204; doi:10.1371/journal.pmed.1004197)

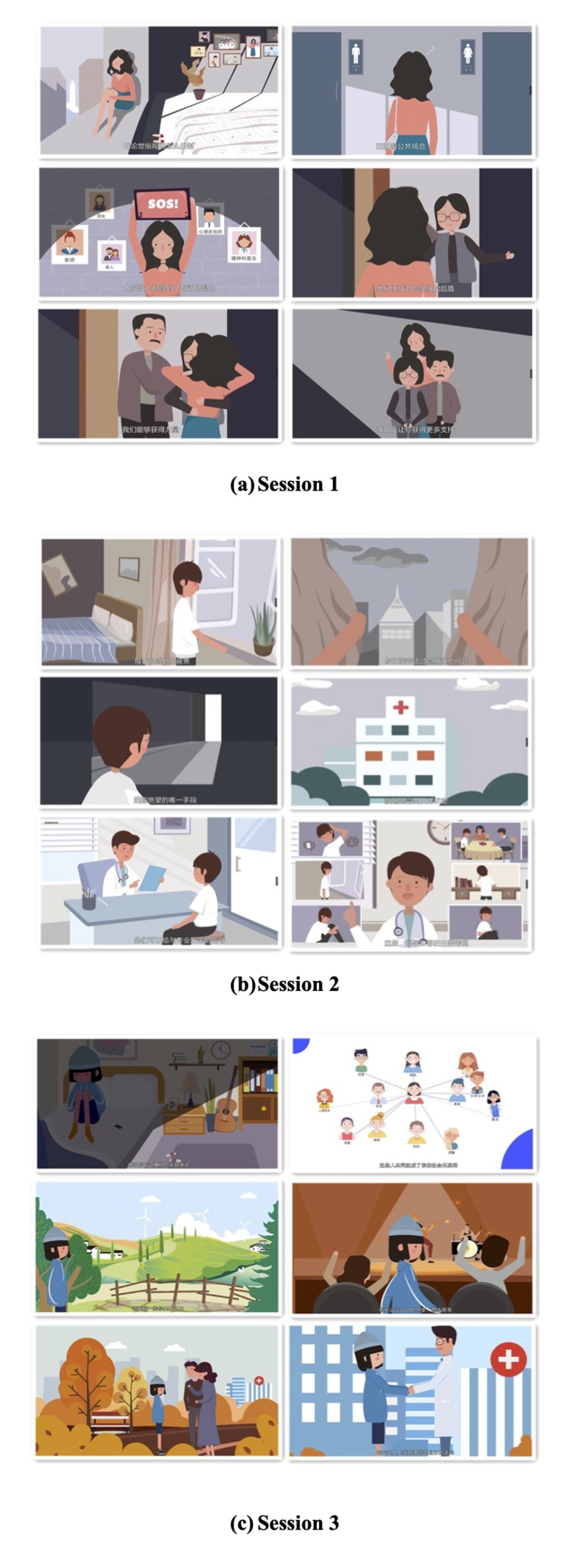

Supplement: S1 Fig — (TIF) [file pmed.1004197.s002.tif]

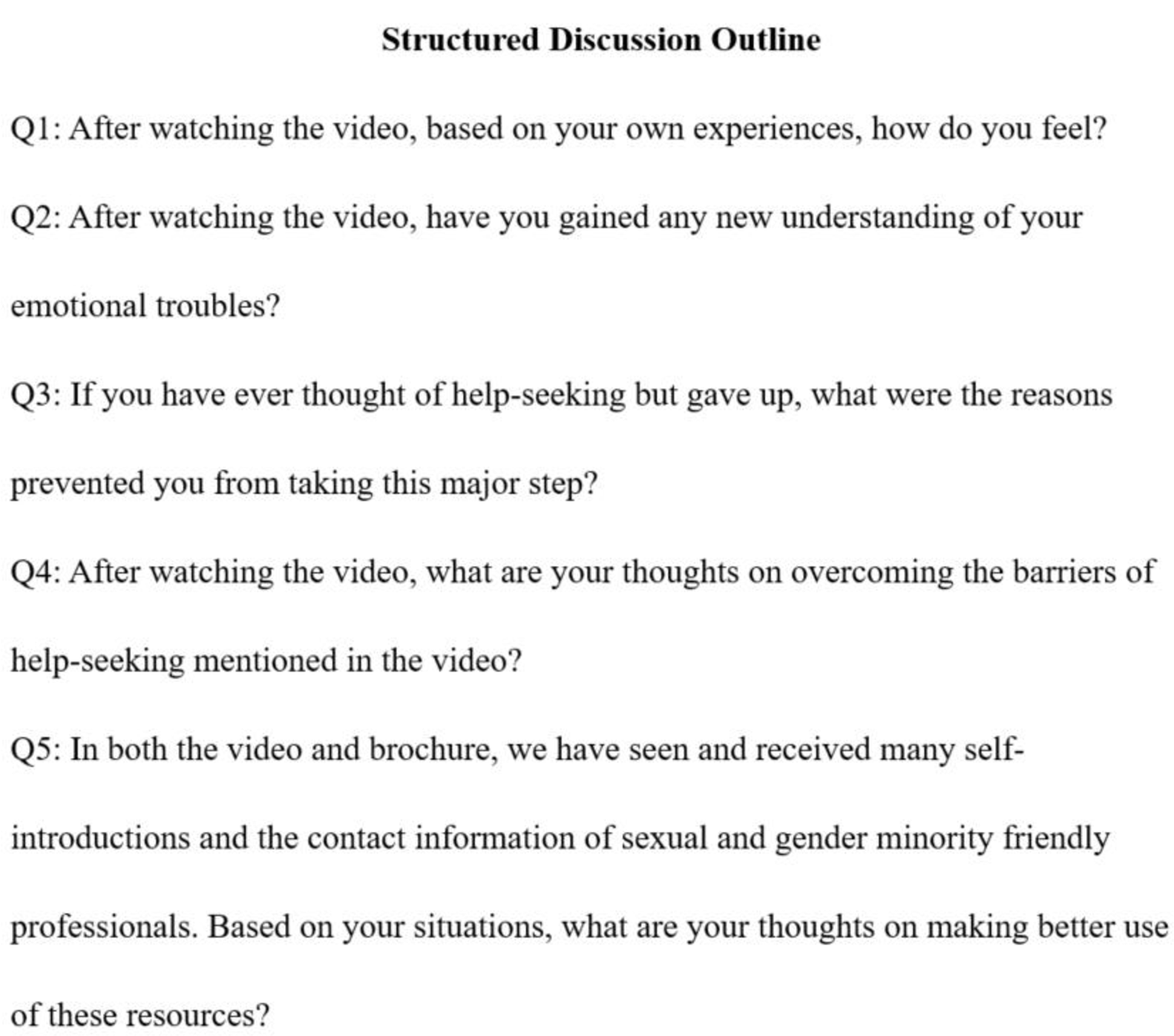

Supplement: S2 Fig — (TIF) [file pmed.1004197.s003.tif]

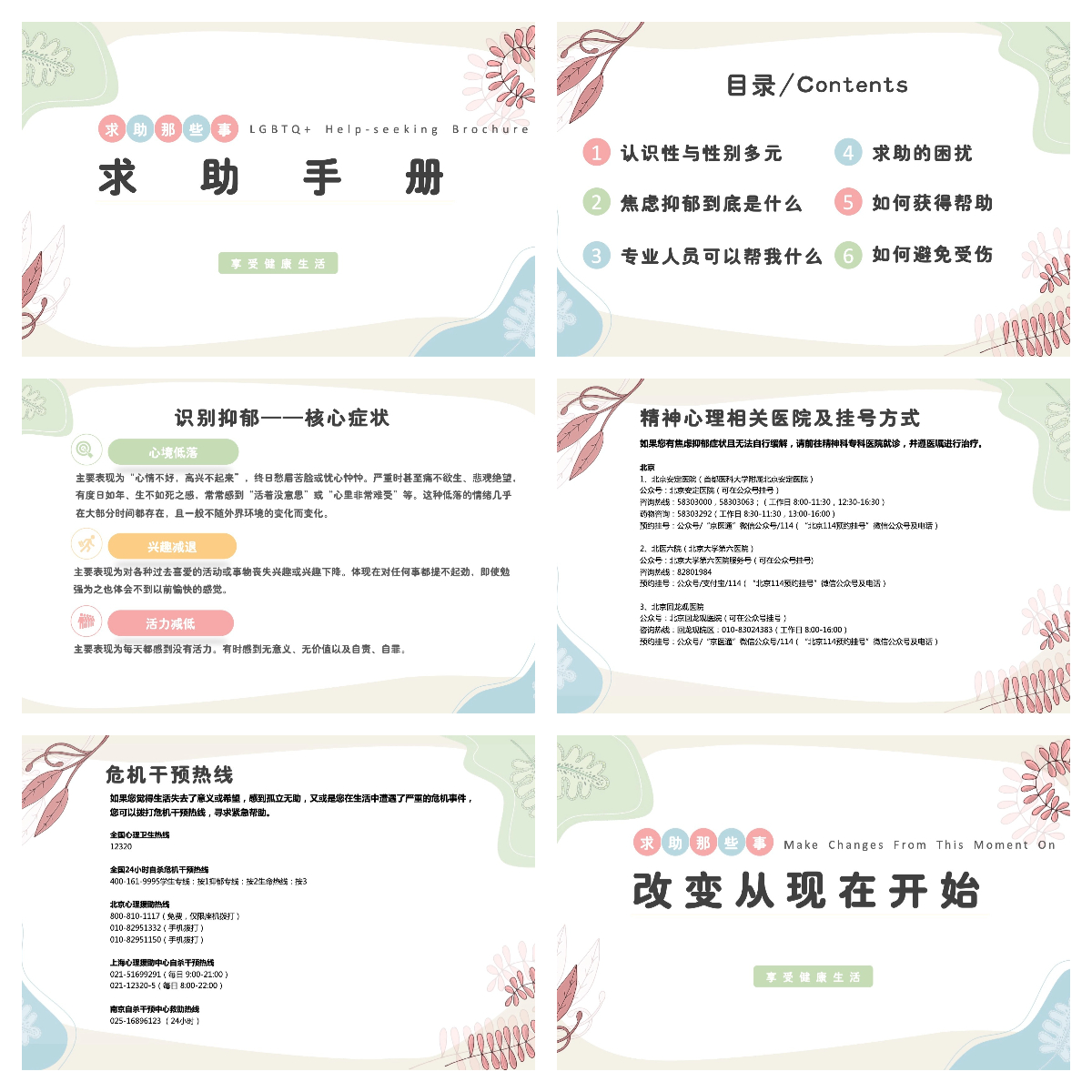

Supplement: S3 Fig — (TIF) [file pmed.1004197.s004.tif]

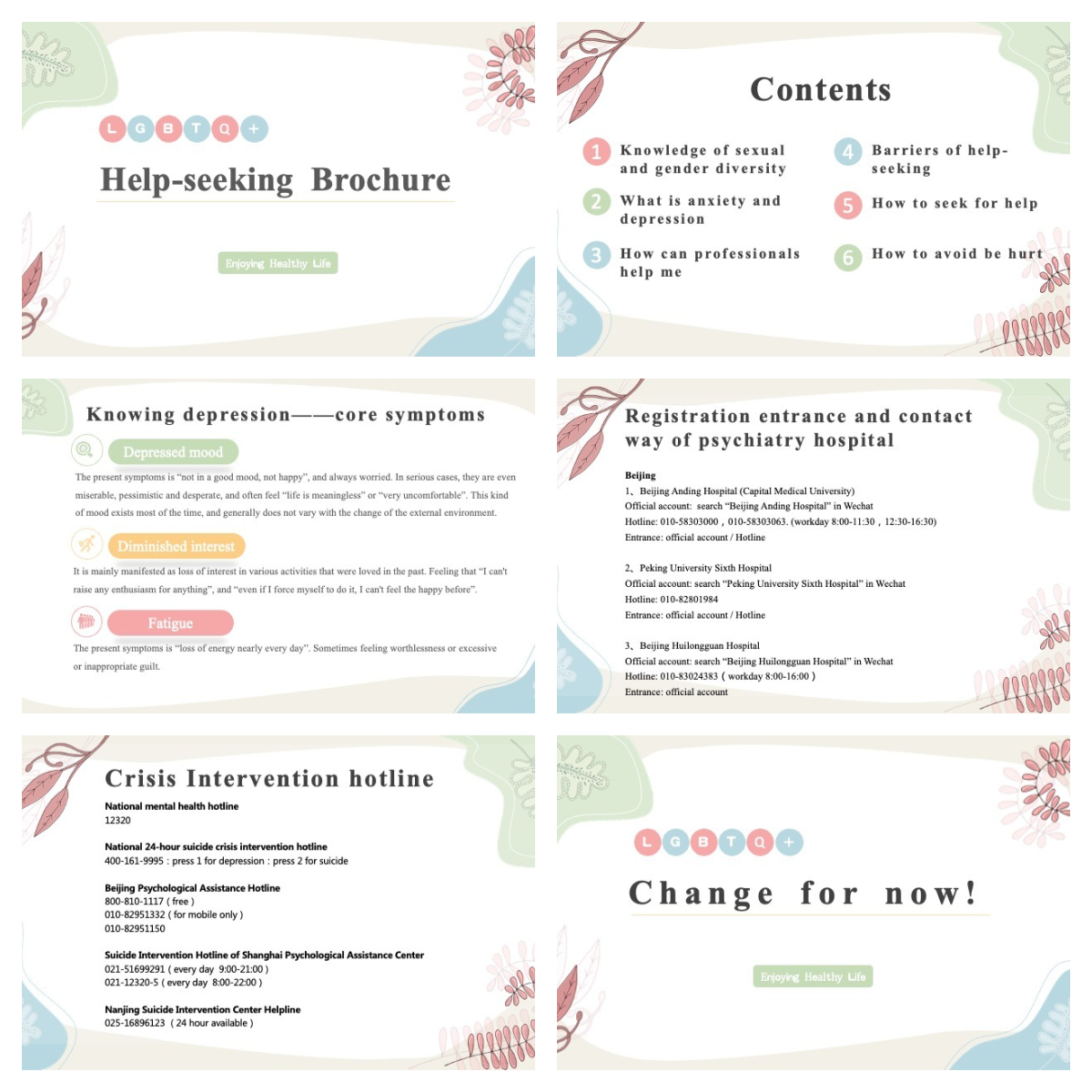

Supplement: S4 Fig — (TIF) [file pmed.1004197.s005.tif]
