# Supplementary material for: A multicomponent digital intervention to promote help-seeking for mental health problems and suicide in sexual and gender diverse young adults: A randomized controlled trial
Source: PLoS Med. 2023 Mar 6;20(3):e1004197. doi: 10.1371/journal.pmed.1004197 (PMC10027204; doi:10.1371/journal.pmed.1004197)
Supplement: S1 Table — (DOCX) [file pmed.1004197.s006.docx]

| **S1 Table. Results from the generalized mixed linear model.** | | | | |
| --- | --- | --- | --- | --- |
|  | Observed estimates | | Model-based estimates | |
|  | Intervention group | Control group | OR (95%CI) | P value |
| **GHSQ-emotion** |  |  |  |  |
| Intimate partner |  |  |  |  |
| Baseline | 34(91.9) | 37(90.2) | - | - |
| Post-intervention | 34(94.4) | 34(94.4) | 0.82(0.06-11.31) | 0.876 |
| 1 month | 34(97.1) | 35(94.6) | 1.59(0.09-46.88) | 0.756 |
| 3 months | 32(94.1) | 32(94.1) | 0.82(0.06-11.33) | 0.876 |
| Parent/family member |  |  |  |  |
| Baseline | 27(40.3) | 30(41.7) | - | - |
| Post-intervention | 30(43.5) | 30(43.5) | 1.06 (0.41-2.75) | 0.908 |
| 1 month | 32(46.4) | 33(47.8) | 1.00 (0.39-2.59) | 0.997 |
| 3 months | 35(52.2) | 32(46.4) | 1.34 (0.52-3.49) | 0.550 |
| Friend |  |  |  |  |
| Baseline | 64(95.5) | 65(91.5) | - | - |
| Post-intervention | 66(95.7) | 61(89.7) | 1.28(0.17-9.98) | 0.807 |
| 1 month | 65(94.2) | 63(92.6) | 0.65(0.08-4.70) | 0.674 |
| 3 months | 58(86.6) | 62(89.9) | 0.37(0.06-2.09) | 0.271 |
| Mental health professional |  |  |  |  |
| Baseline | 56(82.4) | 57(79.2) | - | - |
| Post-intervention | 63(90.0) | 59(86.8) | 1,12(0.29-4.38) | 0.871 |
| 1 month | 62(89.9) | 59(85.5) | 1.22(0.32-4.73) | 0.768 |
| 3 months | 62(91.2) | 63(91.3) | 0.80(0.18-3.48) | 0.765 |
| Psychiatrist |  |  |  |  |
| Baseline | 41(61.2) | 34(47.9) | - | - |
| Post-intervention | 55(78.6) | 41(61.2) | 1.35(0.49-3.76) | 0.557 |
| 1 month | 53(76.8) | 41(60.3) | 1.27(0.47-3.49) | 0.639 |
| 3 months | 56(82.4) | 44(64.7) | 1.48(0.52-4.28) | 0.460 |
| Other Doctors |  |  |  |  |
| Baseline | 15(22.1) | 8(11.3) | - | - |
| Post-intervention | 28(40.0) | 11(16.2) | 1.55(0.45-5.31) | 0.485 |
| 1 month | 21(31.3) | 10(14.7) | 1.19(0.33-4.20) | 0.789 |
| 3 months | 20(30.8) | 14(20.6) | 0.77(0.22-2.59) | 0.674 |
| Phone helpline |  |  |  |  |
| Baseline | 26(38.8) | 30(41.7) | - | - |
| Post-intervention | 46(65.7) | 39(56.5) | 1.66(0.63-4.38) | 0.303 |
| 1 month | 40(58.0) | 37(53.6) | 1.34(0.52-3.50) | 0.545 |
| 3 months | 41(60.3) | 42(60.9) | 1.10(0.42-2.89) | 0.847 |
| Informational websites on the internet |  |  |  |  |
| Baseline | 30(44.8) | 35(48.6) | - | - |
| Post-intervention | 46(66.7) | 40(58.0) | 1.69(0.65-4.44) | 0.284 |
| 1 month | 45(65.2) | 35(50.7) | 2.13(0.82-5.56) | 0.122 |
| 3 months | 48(71.6) | 42(60.9) | 1.89(0.71-5.08) | 0.201 |
| Self-help programs on the internet |  |  |  |  |
| Baseline | 33(48.5) | 36(50.0) | - | - |
| Post-intervention | 45(66.2) | 42(60.9) | 1.33(0.51-3.50) | 0.557 |
| 1 month | 45(66.2) | 36(52.2) | 1.90(0.73-4.98) | 0.188 |
| 3 months | 43(63.2) | 41(59.4) | 1.25(0.48-3.25) | 0.652 |
| Social media |  |  |  |  |
| Baseline | 34(50.7) | 38(52.8) | - | - |
| Post-intervention | 43(62.3) | 40(58.0) | 1.30(0.50-3.38) | 0.589 |
| 1 month | 45(66.2) | 34(49.3) | 2.18(0.84-5.73) | 0.110 |
| 3 months | 41(61.2) | 37(53.6) | 1.48(0.57-3.85) | 0.421 |
| **GHSQ-suicide** |  |  |  |  |
| Intimate partner |  |  |  |  |
| Baseline | 30(78.9) | 32(80.0) | - | - |
| Post-intervention | 32(88.9) | 28(77.8) | 2.44(0.45-14.26) | 0.306 |
| 1 month | 31(96.9) | 28(82.4) | 7.09(0.80-160.12) | 0.116 |
| 3 months | 33(94.3) | 25(73.5) | 6.34(0.99-56.46) | 0.064 |
| Parent/family member |  |  |  |  |
| Baseline | 18(26.5) | 22(31.9) | - | - |
| Post-intervention | 30(44.8) | 25(37.9) | 1.73(0.63-4.79) | 0.289 |
| 1 month | 29(44.3) | 27(40.9) | 1.43(0.52-3.96) | 0.485 |
| 3 months | 34(50.7) | 27(40.3) | 1.98(0.73-5.47) | 0.182 |
| Friend |  |  |  |  |
| Baseline | 50(73.5) | 57(82.6) | - | - |
| Post-intervention | 61(88.4) | 49(75.4) | 4.26(1.26-15.20) | 0.022 |
| 1 month | 57(85.1) | 53(80.3) | 2.39(0.71-8.28) | 0.163 |
| 3 months | 60(89.6) | 53(80.3) | 3.60(1.01-13.51) | 0.052 |
| Mental health professional |  |  |  |  |
| Baseline | 57(85.1) | 56(80.0) | - | - |
| Post-intervention | 64(94.1) | 57(87.7) | 1.58(0.35-7.86) | 0.562 |
| 1 month | 60(89.6) | 54(83.1) | 1.23(0.32-4.83) | 0.768 |
| 3 months | 63(92.6) | 54(81.8) | 1.96(0.49-8.55) | 0.351 |
| Psychiatrist |  |  |  |  |
| Baseline | 52(76.5) | 51(72.9) | - | - |
| Post-intervention | 59(86.8) | 48(75.0) | 1.80(0.56-6.01) | 0.329 |
| 1 month | 56(83.6) | 40(61.5) | 2.63(0.86-8.20) | 0.092 |
| 3 months | 61(89.7) | 42(63.6) | 4.11(1.26-14.28) | 0.022 |
| Other Doctors |  |  |  |  |
| Baseline | 17(25.0) | 11(16.2) | - | - |
| Post-intervention | 27(40.3) | 8(12.3) | 2.78(0.82-9.72) | 0.102 |
| 1 month | 23(34.8) | 15(23.1) | 1.03(0.33-3.24) | 0.957 |
| 3 months | 23(34.3) | 16(24.2) | 0.95(0.30-2.94) | 0.923 |
| Phone helpline |  |  |  |  |
| Baseline | 38(55.9) | 33(47.1) | - | - |
| Post-intervention | 47(69.1) | 39(60.0) | 1.05(0.39-2.81) | 0.921 |
| 1 month | 44(65.7) | 37(56.1) | 1.06(0.40-2.79) | 0.913 |
| 3 months | 44(64.7) | 44(65.7) | 0.67(0.25-1.79) | 0.429 |
| Informational websites on the internet |  |  |  |  |
| Baseline | 29(43.9) | 24(35.3) | - | - |
| Post-intervention | 37(55.2) | 35(53.8) | 0.74(0.28-1.95) | 0.538 |
| 1 month | 36(53.7) | 33(50.0) | 0.81(0.30-2.14) | 0.668 |
| 3 months | 44(65.7) | 39(58.2) | 0.96(0.36-2.57) | 0.929 |
| Self-help programs on the internet |  |  |  |  |
| Baseline | 24(35.3) | 26(37.1) | - | - |
| Post-intervention | 37(54.4) | 31(47.7) | 1.42(0.54-3.77) | 0.482 |
| 1 month | 36(54.5) | 29(43.9) | 1.66(0.63-4.42) | 0.310 |
| 3 months | 41(60.3) | 36(53.7) | 1.42(0.54-3.77) | 0.484 |
| Social media |  |  |  |  |
| Baseline | 30(44.1) | 30(44.1) | - | - |
| Post-intervention | 40(58.8) | 29(45.3) | 1.72(0.66-4.55) | 0.269 |
| 1 month | 39(59.1) | 32(48.5) | 1.53(0.59-4.04) | 0.385 |
| 3 months | 37(55.2) | 34(50.7) | 1.20(0.46-3.13) | 0.713 |
| **AHSQ** |  |  |  |  |
| Intimate partner |  |  |  |  |
| Baseline | 32(80.0) | 29(80.6) | - | - |
| 1 month | 34(85.0) | 24(77.4) | 1.71(0.33-9.20) | 0.525 |
| 3 months | 31(79.5) | 26(70.3) | 1.70(0.36-8.13) | 0.501 |
| Parent/family member |  |  |  |  |
| Baseline | 43(62.3) | 35(49.3) | - | - |
| 1 month | 40(58.0) | 39(57.4) | 0.60(0.23-1.57) | 0.229 |
| 3 months | 40(58.8) | 31(45.6) | 1.00(0.38-2.61) | 0.996 |
| Friend |  |  |  |  |
| Baseline | 59(85.5) | 63(87.5) | - | - |
| 1 month | 62(89.9) | 61(88.4) | 1.38(0.32-5.97) | 0.664 |
| 3 months | 63(92.6) | 58(84.1) | 2.84(0.66-13.11) | 0.167 |
| Mental health professional |  |  |  |  |
| Baseline | 28(41.2) | 30(42.3) | - | - |
| 1 month | 27(39.7) | 32(47.8) | 0.75(0.29-1.97) | 0.562 |
| 3 months | 31(46.3) | 33(49.3) | 0.93(0.36-2.42) | 0.877 |
| Psychiatrist |  |  |  |  |
| Baseline | 16(23.5) | 11(15.5) | - | - |
| 1 month | 16(23.2) | 14(20.9) | 0.68(0.21-2.21) | 0.522 |
| 3 months | 17(25.4) | 14(21.2) | 0.75(0.23-2.43) | 0.635 |
| Other Doctors |  |  |  |  |
| Baseline | 5(7.5) | 3(4.2) | - | - |
| 1 month | 5(7.2) | 6(9.0) | 0.43(0.06-2.91) | 0.396 |
| 3 months | 7(10.6) | 4(6.1) | 1.01(0.13-7.21) | 0.995 |
| Phone helpline |  |  |  |  |
| Baseline | 12(17.6) | 7(9.7) | - | - |
| 1 month | 9(13.0) | 13(19.1) | 0.32(0.08-1.22) | 0.10 |
| 3 months | 15(22.1) | 13(19.1) | 0.60(0.16-2.19) | 0.44 |
| Informational websites on the internet |  |  |  |  |
| Baseline | 22(32.8) | 18(25.0) | - | - |
| 1 month | 32(46.4) | 21(30.9) | 1.32(0.48-3.66) | 0.593 |
| 3 months | 29(42.6) | 26(38.2) | 0.82(0.30-2.24) | 0.698 |
| Self-help programs on the internet |  |  |  |  |
| Baseline | 26(38.2) | 25(34.7) | - | - |
| 1 month | 31(45.6) | 23(33.3) | 1.44(0.54-3.84) | 0.464 |
| 3 months | 30(44.1) | 24(35.3) | 1.24(0.47-3.31) | 0.661 |
| Social media |  |  |  |  |
| Baseline | 32(48.5) | 26(36.6) | - | - |
| 1 month | 35(50.7) | 22(31.9) | 1.35(0.51-3.58) | 0.545 |
| 3 months | 28(41.8) | 24(35.3) | 0.81(0.30-2.14) | 0.668 |
| Data are n (%), valid percentage.  In all variables showed in this table, higher values correspond to better outcomes.  OR was calculated using post hoc analysis from the generalized linear mixed model, and follow-up percentage were compared after subtracting the baseline percentage of both groups separately.  GHSQ = General Help-Seeking Questionnaire; AHSQ = Actual Help Seeking Questionnaire. | | | | |
