# Supplementary material for: A multicomponent digital intervention to promote help-seeking for mental health problems and suicide in sexual and gender diverse young adults: A randomized controlled trial
Source: PLoS Med. 2023 Mar 6;20(3):e1004197. doi: 10.1371/journal.pmed.1004197 (PMC10027204; doi:10.1371/journal.pmed.1004197)
Supplement: S2 Table — (DOCX) [file pmed.1004197.s007.docx]

| **S2 Table. Sensitivity Analyses of the linear mixed model.** | | | | |
| --- | --- | --- | --- | --- |
|  | Intervention condition | Control  condition | Mean difference ^a^ (95%CI) | P value |
|  | Mean (95CI%) | Mean (95CI%) |  |  |
| **Primary outcomes** |  |  |  |  |
| **GHSQ** |  |  |  |  |
| GHSQ-emotion score ^b, c^ |  |  |  |  |
| Baseline | 2.69(2.57,2.80) | 2.62(2.51,2.73) | - | - |
| Post-discussion | 2.93(2.82,3.04) | 2.76(2.65,2.87) | 0.11(-0.01,0.22) | 0.220 ^f^ |
| 1 month ^e^ | 2.91(2.79,3.02) | 2.69(2.58,2.81) | 0.15(0.03,0.27) | 0.039 ^f^ |
| 3 months | 2.96(2.84,3.07) | 2.76(2.65,2.87) | 0.13(0.01,0.25) | 0.088 ^f^ |
| GHSQ-suicide score ^b, c^ |  |  |  |  |
| Baseline | 2.57(2.44,2.70) | 2.58(2.45,2.71) | - | - |
| Post-discussion | 2.91(2.78,3.04) | 2.69(2.56,2.83) | 0.23(0.08,0.38) | 0.008 ^f^ |
| 1 month ^e^ | 2.87(2.74,3.00) | 2.68(2.55,2.82) | 0.20(0.04,0.35) | 0.035 ^f^ |
| 3 months | 2.95(2.81,3.08) | 2.70(2.57,2.83) | 0.25(0.10,0.40) | 0.003 ^f^ |
| **ATSPPH-SF score** ^c^ |  |  |  |  |
| Baseline | 18.60(17.55,19.64) | 19.29(18.27,20.32) | - | - |
| Post-discussion | 19.99(18.95,21.03) | 21.18(20.14,22.22) | -0.50(-1.80,0.79) | 1.000 ^f^ |
| 1 month ^e^ | 20.75(19.71,21.79) | 21.18(20.14,22.22) | 0.26(-1.04,1.56) | 1.000 ^f^ |
| 3 months | 21.64(20.59,22.69) | 21.43(20.39,22.47) | 0.90(-0.40,2.21) | 0.525 ^f^ |
| **Secondary outcome** |  |  |  |  |
| **AHSQ score** ^b, c^ |  |  |  |  |
| Baseline | 0.46(0.41,0.51) | 0.41(0.36,0.45) | - | - |
| 1 month ^e^ | 0.48(0.43,0.53) | 0.44(0.39,0.49) | -0.02(-0.07,0.04) | 0.582 |
| 3 months | 0.49(0.44,0.54) | 0.44(0.39,0.49) | -0.01(-0.06,0.05) | 0.795 |
| **Depression-Anxiety-Lit** |  |  |  |  |
| Depression-Lit score ^c^ |  |  |  |  |
| Baseline | 13.33(12.47,14.19) | 12.90(12.05,13.75) | - | - |
| Post-discussion | 13.76(12.90,14.62) | 12.19(11.34,13.05) | 1.14(0.24,2.04) | 0.014 |
| 1 month ^e^ | 14.26(13.39,15.12) | 12.03(11.18,12.89) | 1.80(0.90,2.70) | <0.001 |
| 3 months | 13.90(13.03,14.76) | 12.00(11.15,12.86) | 1.47(0.56,2.37) | 0.002 |
| Anxiety-Lit score ^c^ |  |  |  |  |
| Baseline | 12.04(11.25,12.82) | 12.04(11.27,12.81) | - | - |
| Post-discussion | 13.37(12.59,14.15) | 11.86(11.08,12.64) | 1.51(0.59,2.43) | 0.001 |
| 1 month ^e^ | 13.92(13.14,14.70) | 11.95(11.17,12.73) | 1.97(1.05,2.89) | <0.001 |
| 3 months | 13.85(13.06,14.63) | 11.92(11.14,12.70) | 1.93(1.01,2.85) | <0.001 |
| **SSOSH score** ^d^ |  |  |  |  |
| Baseline | 24.09(22.73,25.45) | 23.17(21.83,24.51) | - | - |
| Post-discussion | 23.19(21.83,24.54) | 21.95(20.59,23.30) | 0.32(-1.15,1.79) | 0.670 |
| 1 month ^e^ | 23.23(21.87,24.58) | 23.08(21.72,24.43) | -0.77(-2.24,0.70) | 0.305 |
| 3 months | 22.91(21.55,24.27) | 22.26(20.91,23.62) | -0.28(-1.75,1.20) | 0.714 |
| **HSERK score** ^c^ |  |  |  |  |
| Baseline | 32.91(31.91,33.91) | 33.19(32.21,34.18) | - | - |
| Post-discussion | 34.36(33.36,35.35) | 33.20(32.21,34.20) | 1.44(0.15,2.73) | 0.029 |
| 1 month ^e^ | 34.29(33.29,35.29) | 33.03(32.03,34.03) | 1.54(0.25,2.84) | 0.020 |
| 3 months | 35.00(33.99,36.00) | 33.48(32.48,34.48) | 1.80(0.51,3.10) | 0.007 |
| **DASS21** |  |  |  |  |
| DASS21-depreesion score ^d^ |  |  |  |  |
| Baseline | 19.27(16.86,21.69) | 17.06(14.68,19.43) | - | - |
| Post-discussion | 18.00(15.59,20.41) | 16.01(13.60,18.41) | -0.23(-2.99,2.54) | 0.873 |
| 1 month ^e^ | 16.34(13.92,18.76) | 14.36(11.95,16.76) | -0.23(-3.01,2.54) | 0.870 |
| 3 months | 17.94(15.51,20.36) | 17.14(14.73,19.54) | -1.42(-4.20,1.36) | 0.317 |
| DASS21-anxiety score ^d^ |  |  |  |  |
| Baseline | 16.26(14.18,18.34) | 13.94(11.90,15.99) | - | - |
| Post-discussion | 16.29(14.21,18.36) | 14.44(12.37,16.51) | -0.47(-2.98,2.05) | 0.718 |
| 1 month ^e^ | 13.92(11.84,16.01) | 12.67(10.60,14.74) | -1.06(-3.58,1.47) | 0.412 |
| 3 months | 15.21(13.12,17.30) | 14.44(12.37,16.51) | -1.54(-4.07,0.99) | 0.233 |
| DASS21-stress score ^d^ |  |  |  |  |
| Baseline | 22.68(20.42,24.95) | 20.64(18.41,22.87) | - | - |
| Post-discussion | 21.60(19.34,23.86) | 20.59(18.33,22.85) | -1.03(-4.03,1.96) | 0.499 |
| 1 month ^e^ | 20.34(18.08,22.61) | 17.49(15.23,19.75) | 0.81(-2.19,3.82) | 0.596 |
| 3 months | 20.96(18.68,23.24) | 20.27(18.01,22.53) | -1.36(-4.37,1.66) | 0.378 |
| GHSQ = General Help-Seeking Questionnaire; ATSPPH-SF = Attitudes Toward Seeking Professional Psychological Help Scale-Short Form; AHSQ = Actual Help-Seeking Questionnaire; Depression-Lit = Depression Literacy Questionnaire; Anxiety-Lit = Anxiety Literacy Questionnaire; SSOSH = Self-Stigma of Seeking Help Scale; HSERK = Help-Seeking Encouragement Related Knowledge Scale; DASS21= Depression Anxiety and Stress Scale 21.  ^a^ Mean difference was calculated using post hoc analysis from the linear mixed model, and follow-up scores were compared after subtracting the baseline scores of both groups separately.  ^b^ Some participants chose ‘not applicable’ in the whole questionnaire.  ^c^ Higher values correspond to better outcomes.  ^d^ Higher values correspond to worse outcomes.  ^e^ Primary timepoint.  ^f^ P value was adjusted by the Bonferroni-corrected method (3🞨P value), level of significance still was P < .05. | | | | |
