# Supplementary material for: A multicomponent digital intervention to promote help-seeking for mental health problems and suicide in sexual and gender diverse young adults: A randomized controlled trial
Source: PLoS Med. 2023 Mar 6;20(3):e1004197. doi: 10.1371/journal.pmed.1004197 (PMC10027204; doi:10.1371/journal.pmed.1004197)
Supplement: S3 Table — (DOCX) [file pmed.1004197.s008.docx]

| **S3 Table. Results of the intervention evaluation.** | | | | |
| --- | --- | --- | --- | --- |
|  | Intervention group  (n = 70) | Control group  (n = 69) | *t*-test | P value |
| Video |  |  |  |  |
| Quality | 4.06(0.72) | 3.94(0.68) | 0.97 | 0.336 |
| Involvement | 4.00(0.76) | 3.88(0.74) | 0.91 | 0.364 |
| Satisfaction | 3.93(0.73) | 3.96(0.74) | -0.23 | 0.822 |
| Group discussion |  |  |  |  |
| Involvement | 4.16(0.85) | 4.09(0.72) | 0.53 | 0.599 |
| Satisfaction | 4.09(0.78) | 4.06(0.64) | 0.23 | 0.818 |
| Brochure^*^ |  |  |  |  |
| Quality | 3.94(0.73) | 3.77(0.86) | 1.28 | 0.201 |
| Involvement | 3.43(0.90) | 3.33(1.07) | 0.60 | 0.547 |
| Satisfaction | 3.87(0.82) | 3.70(0.86) | 1.21 | 0.227 |
| Entire invention^*^ |  |  |  |  |
| Involvement | 3.64(0.69) | 3.59(0.79) | 0.35 | 0.731 |
| Satisfaction | 3.83(0.71) | 3.74(0.90) | 0.63 | 0.529 |
| Data are mean (SD). ^*^One participant in the intervention group had missing data for these variables. | | | | |
