# Supplementary material for: A multicomponent digital intervention to promote help-seeking for mental health problems and suicide in sexual and gender diverse young adults: A randomized controlled trial
Source: PLoS Med. 2023 Mar 6;20(3):e1004197. doi: 10.1371/journal.pmed.1004197 (PMC10027204; doi:10.1371/journal.pmed.1004197)
Supplement: S1 Text — (DOCX) [file pmed.1004197.s009.docx]

**S1 Text. Items to include when reporting a randomized trial in a journal or conference abstract**

| **Item** | **Description** | **Reported on line number** |
| --- | --- | --- |
| Title | Identification of the study as randomized | 1-3 |
| Authors * | Contact details for the corresponding author | 5-7 |
| Trial design | Description of the trial design (e.g. parallel, cluster, non-inferiority) | 39 |
| Methods |  |  |
| Participants | Eligibility criteria for participants and the settings where the data were collected | 35-37 |
| Interventions | Interventions intended for each group | 43-45 |
| Objective | Specific objective or hypothesis | 31-33 |
| Outcome | Clearly defined primary outcome for this report | 45-47 |
| Randomization | How participants were allocated to interventions | 38-40 |
| Blinding (masking) | Whether or not participants, care givers, and those assessing the outcomes were blinded to group assignment | 40 |
| Results |  |  |
| Numbers randomized | Number of participants randomized to each group | 38-39 |
| Recruitment | Trial status | 35;42-43 |
| Numbers analysed | Number of participants analysed in each group | 53-54 |
| Outcome | For the primary outcome, a result for each group and the estimated effect size and its precision | 55-57 |
| Harms | Important adverse events or side effects | 64-65 |
| Conclusions | General interpretation of the results | 69-72 |
| Trial registration | Registration number and name of trial register | 50 |
| Funding | Source of funding | N.A. |

**this item is specific to conference abstracts*
