# Supplementary material for: A multicomponent digital intervention to promote help-seeking for mental health problems and suicide in sexual and gender diverse young adults: A randomized controlled trial
Source: PLoS Med. 2023 Mar 6;20(3):e1004197. doi: 10.1371/journal.pmed.1004197 (PMC10027204; doi:10.1371/journal.pmed.1004197)
Supplement: S1 Protocol — (DOCX) [file pmed.1004197.s010.docx]

## S1 Protocol

Promote help-seeking for mental health in LGBTQ+ young adults: Protocol for a randomized controlled trial

Dr Runsen Chen, Tsinghua University, Principal Investigator

Trial registration number: ChiCTR2100053248

Version: 1.0

Data: 2021/10/20

**1 Study objectives**

**1.1 Primary Aim**

The primary objective of this study is to investigate the efficacy of an integrative internet-based psychoeducational help-seeking intervention in improving the help-seeking intention and attitude of the sexual and gender minority (SGM) population. Changes in help-seeking intention and attitude will be assessed by comparing the intervention and control conditions at base line and post-intervention, 1-month and 3-month post intervention follow-up.

**1.2 Secondary Aim**

The second objective of this study is to test the efficacy of the intervention on secondary outcomes, including actual help-seeking behaviors, depression and anxiety literacy, help-seeking stigma, and help-seeking encouragement related knowledge.

**1.3 Hypotheses**

H1: Participants in the intervention condition will report greater improvements in help-seeking intention and attitude compared to the control condition.

H2: Participants in the intervention condition will report greater improvements in internalized help-seeking stigma, help-seeking literacy, mental health literacy and help-seeking behaviors compared to the control condition.

1. **Study Background**

**2.1 Rationale**

The sexual and gender minority young adults, compared to its cis-heterosexual peers, has been facing elevated risks for mental health concerns. Despite their higher susceptibility to mental health risks, compared to their cis-heterosexual peers, they have been shown to be less inclined and likely to seek help under the impact of minority stress, help-seeking stigma, and lower mental health literacy. The combined effects of these factors further restrained SGM young adults’ help-seeking intentions, attitudes, and behaviors. Nevertheless, there is a paucity of research concentrated on improving SGM young adults’ help-seeking. The effectiveness of psychoeducational videos, facilitator-led group discussions, and help-seeking brochures have been separately demonstrated in previous studies. However, there has been no study that utilized an integrated approach combining these three therapeutic elements into one single intervention, leaving a gap in the literature the present study aimed to fill.

**2.2 The help-seeking intervention study**

The help-seeking intervention study aims to evaluate whether the internet-based help-seeking program including intervention video, online structured discussion and electronic brochure is effective for improving help-seeking intention and attitude in sexual and gender minority young adults with mental health problems. The findings of this study will provide more evidence for using digital intervention to improve sexual and gender minority young adults to seek help for mental health problems.

1. **Study Design**

**3.1 Trail design**

We will conduct a randomized controlled trial (RCT) design to evaluate the efficacy of an internet-based help-seeking program promoting the intention and attitude of help-seeking at sexual and gender minority young adults. Researchers not directly involved in this study contribute to the randomization process, ensuring its validity.

**3.2 Participants**

144 sexual and gender minority young adult participants will be recruited and randomly allocated (1:1) to the experimental condition and control condition.

**3.2.1 Inclusion criteria**

Participants need to meet the following criteria to be enrolled in the current experiment:

1. Age between 18-29 years old.
2. Identify themselves as sexual and gender minority.
3. The DASS-21 scale score is moderate or above in at least one dimension.
4. Living in the People’s Republic of China.
5. Have stable internet connections.
6. Must complete all assessments and interventions required in the research.

**3.2.2 Exclusion criteria**

Participants are excluded from enrollment if they satisfy any of the following criteria:

1. History of being diagnosed with a severe psychotic disorder, such as schizophrenia.
2. Have had suicidal attempt(s) in the last six months.
3. Currently have severe suicidal ideations.
4. Have help-seeking experience(s) from mental health professionals in the past 12 months.
5. Refuse to sign the consent form or are unwilling to participate in the current project.
6. Do not have access to a quiet, undisturbed space that has stable internet.
7. **Sample size**

The sample size was calculated based on the expected effects of the intervention on the primary outcome measures (i.e., help-seeking intentions and attitudes). Given the moderate effect sizes of help-seeking intentions (i.e., Cohen’s d = 0.53) and help-seeking attitudes (i.e., Cohen’s d = 0.58) found in a previous study, researchers conservatively estimated a sample size consisting of 144 participants in total, assuming 20% attrition and 1:1 allocation ratio during randomization. This sample size would provide 80% power to detect an effect size of 0.53 at the α level of 0.05 for each of the two primary outcomes.

1. **Procedures**

Participants were recruited online in the first stage. Those who met the inclusion criteria need to complete the baseline questionnaire and online experiment tasks. Subsequently, one week after baseline collection, all participants were randomly allocated into either the experimental or control group via a random number table by a researcher who is not responsible for recruiting and data collection for this current project. All research facilitators who lead the experimental group were professionally trained.

Each experimental or control group, consisting of approximately 4-6 participants, watched the intervention video (a deliberately edited video that contains information about mental health, self-harm, suicide, help-seeking tips, and the mental health professionals’ self-introduction) or the control video (animation about healthy sleep knowledge). Immediately after watching the video, all group members were guided by their research facilitator and engage in a group (45 in time length, audio recorded). A questionnaire was given at the end of the discussion.

Within two weeks after the first session, participants could watch the intervention video as many times as they prefer, and the duration and number of viewings were recorded. The digital sexual-and-gender-minority-friendly brochures were also available to participants. Two follow-up surveys will be given at 4 weeks post-intervention and 12 weeks post-intervention, respectively.

1. **Statistical analysis**

Researchers used the intention-to-treat analytic approach to process and analyze data from the following time points: (1) post-intervention, (2) 1-month post-intervention, and (3) 3-month post-intervention. Researchers analyzed the actual help-seeking behaviors of participants by analyzing only data collected from 1-month post-intervention and 3-month post-intervention while considering actual situations in which they seek help. Researchers used the Linear Mixed Model (LMM) and the Generalized Linear Mixed Model (GLMM) for statistical analysis. The significance level of each analysis was set to 0.05 while simultaneously reporting a 95% confidence interval.

1. **Privacy**

We kept the participants’ research records confidential as required by law and the ethics committee at Tsinghua University, which provides guarantees for privacy security, data protection, and authorized access. Unless required by relevant law enforcement, participants’ names, national identification numbers, addresses, mobile numbers, or any information that may reveal their identities will not be disclosed outside the study. Participants were identified via a unique made-up ID number given by the researchers at the beginning of the study. Recorded audio information has been immediately deleted after finalizing the content analysis to prevent any information leak. Participants’ identities remained undisclosed for scientific conferences or publications.

1. **Funding source**

This study was supported by The Second Xiangya Hospital, Central South University, National Clinical Research Centre on Mental Disorders and Research Fund, Vanke School of Public Health, Tsinghua University.

1. **Primary outcome questionnaires**

**9.1 General Help-Seeking Questionnaire (GHSQ)**

Subscale (1) If you were having a personal or emotional problem, how likely is it that you would seek help from the following sources?

Subscale (2) If you were experiencing suicidal thoughts, how likely is it that you would seek help from the following source?

Responses: (0) extremely unlikely (1) unlikely (2) likely (3) extremely likely

(NA) Not applicable

| 1 | intimate partner (e.g., girlfriend, boyfriend, husband, wife, de facto) |
| --- | --- |
| 2 | Parent/family member |
| 3 | Friend (not related to you) |
| 4 | Mental health professional (e.g., psychologist, counselor) |
| 5 | Psychiatrist |
| 6 | Other Doctors |
| 7 | Phone helpline (e.g., Lifeline) |
| 8 | Informational websites on the internet |
| 9 | Self-help programs on the internet (e.g., QQ group, Baidu post bar, Douban) |
| 10 | Social media (e.g., Weibo, People network) |
| 11 | I would not seek help from anyone |

**9.2 Attitudes Toward Seeking Professional Help Short Form (ATSPPH-SF)**

Read each statement carefully and indicate your degree of agreement using the scale below. In responding, please be completely candid.

Responses: (0) Disagree (1) Partly disagree (2) Partly agree (3) Agree

| **1** | If I believed I was having a mental breakdown, my first inclination would be to get professional attention. |
| --- | --- |
| **2** | The idea of talking about problems with a psychologist strikes me as a poor way to get rid of emotional conflicts. |
| **3** | If I were experiencing a serious emotional crisis at this point in my life, I would be confident that I could find relief in psychotherapy. |
| **4** | There is something admirable in the attitude of a person who is willing to cope with his or her conflicts and fears without resorting to professional help. |
| **5** | I would want to get psychological help if I were worried or upset for a long period of time. |
| **6** | I might want to have psychological counseling in the future. |
| **7** | A person with an emotional problem is not likely to solve it alone; he or she is likely to solve it with professional help. |
| **8** | Considering the time and expense involved in psychotherapy, it would have doubtful value for a person like me. |
| **9** | A person should work out his or her own problems; getting psychological counseling would be a last resort |
| **10** | Personal and emotional troubles, like many things, tend to work out by themselves |

1. **Secondary and additional outcomes**

**10.1 The Actual Help Seeking Questionnaire (AHSQ)**

The Actual Help Seeking Questionnaire (AHSQ) is used to examine participants' intentions to seek assistance from informal (i.e., friends, family members, online platforms, or self-help resources) and formal sources (i.e., mental health counselors, psychologists, or psychiatrists). For this study, researchers utilize a validated Chinese version of the AHSQ. The scale consists of 11 items on a “Yes”, “No”, or “Not applicable” response scale, with an increasing number of “Yes” representing more help-seeking sources such as the internet, parents, and mental health professionals.

**10.2 Depression and Anxiety Literacy Questionnaire (D-A-Lit)**

The Depression and Anxiety Literacy Questionnaire (D-A-Lit) scale combines the Depression Literacy Questionnaire (D-Lit) and the Anxiety Literacy Questionnaire (A-Lit). The combined scale consists of 44 items, with options including “right,” “wrong,” or “unsure.” Higher scores on this scale represent more knowledge participants have regarding anxiety and depression.

**10.3 Self-Stigma of Seeking Help (SSOSH) Scale**

The Self-Stigma of Seeking Help (SSOSH) scale is designed to measure participants' self-stigma toward seeking professional assistance. For this study, researchers utilize a validated Chinese version of the SSOSH scale. The SSOSH scale consists of 10 items on a 5-point Likert scale, with a higher score indicating a higher level of self-stigma toward help-seeking.

**10.4 Help-Seeking Encouragement Related Knowledge Scale**

The Help-Seeking Encouragement Related Knowledge Scale is used to measure participants' knowledge in help-seeking. For this study, researchers utilize a validated Chinese version of the Help-Seeking Encouragement Related Knowledge Scale. The scale consists of 8 items on a 5-point response scale.

**10.5 Depression Anxiety and Stress Scale-21 (DASS-21)**

Depression Anxiety and Stress Scale 21 (DASS-21) was selected as an essential element in the inclusion criteria. The DASS-21 scale has been validated by previous research in its efficacy, reliability, and validity in measuring dimensions of anxiety, depression, and stress. For this study, researchers utilize a validated Chinese version of the DASS-21. This dimensional self-report scale consists of three subscales. Each subscale consists of 7 items on a 4-point Likert scale, with a higher score indicating a higher level of depression, anxiety, or stress.

1. **Outcome summary**

**Table 1.** summary of primary, secondary, and additional outcomes’ measure.

| Outcomes measures |  | Baseline | Post-intervention | One-month follow-up | Three-month follow-up |
| --- | --- | --- | --- | --- | --- |
| Primary outcome |  |  |  |  |  |
| Help-seeking intentions and attitude | The General Help Seeking Questionnaire (GHSQ) |  |  |  |  |
|  | GHSQ-emotional problems subscale | √ | √ | √ | √ |
|  | GHSQ-suicidal ideation subscale | √ | √ | √ | √ |
|  | The Attitudes Toward Seeking Professional Psychological Help  Scale-Short Form (ATSPPH-SF) | √ | √ | √ | √ |
|  |  |  |  |  |  |
| Secondary outcomes |  |  |  |  |  |
| Help-seeking behaviors | The Actual Help Seeking Questionnaire (AHSQ) | √ |  | √ | √ |
| Depression and anxiety literacy | Depression and Anxiety Literacy Questionnaire (D-A-Lit) | √ | √ | √ | √ |
| Self-stigma of seeking help | Self-Stigma of Seeking Help Scale (SSOHS) | √ | √ | √ | √ |
| Help-seeking encouragement related  knowledge | Help-seeking encouragement related knowledge scale | √ | √ | √ | √ |
|  |  |  |  |  |  |
| Additional outcomes |  |  |  |  |  |
| Depression, anxiety, and stress | Depression Anxiety and Stress Scale-21 (DASS-21) | √ | √ | √ | √ |

| 1. **Investigators training** |
| --- |

| **Training type**  **（Internal or External）** | **Training content** |
| --- | --- |
| Internal | Interview of suicide risk |
| Internal | Training of screening interview |
| Internal | Practice of screening interview |
| Internal | Training of informed consent |
| Internal | Training facilitators how to conduct the intervention process |
| Internal | Discuss the questions encountered in the screening interview |
| Internal | Training facilitators how to lead group discussion |
